# Efficacy and safety of Chinese botanical drug Si Shen Wan in irritable bowel syndrome: a meta-analysis and trial sequential analysis of randomized controlled trials

**DOI:** 10.3389/fphar.2025.1534904

**Published:** 2025-06-02

**Authors:** Qian Liu, Zongming Shi, Yang Jiang, Tao Zhang, SiJing Du, Yemei Gao

**Affiliations:** ^1^ Beijing Jishuitan Hospital, Capital Medical University, Beijing, China; ^2^ Peking University First Hospital, Beijing, China; ^3^ Wangjing Hospital, China Academy of Chinese Medical Sciences, Beijing, China

**Keywords:** Si-Shen-Wan, irritable bowel syndrome, meta-analysis, trial sequential analysis, Chinese botanical drug

## Abstract

**Background and Objectives:**

Irritable bowel syndrome (IBS) is one of the most common functional gastrointestinal disorders (FGIDs), characterized by complex pathogenesis, prolonged disease duration, frequent recurrence, and a significant impact on patients’ quality of life. Si-Shen-Wan (SSW), a renowned traditional Chinese medicine formula, is widely recognized for its efficacy in managing gastrointestinal symptoms, particularly diarrhea, and is commonly used to treat diarrhea-predominant IBS (IBS-D). This study utilized a meta-analysis to evaluate the efficacy and safety of SSW in the treatment of IBS-D.

**Methods:**

A comprehensive search for randomized controlled trials (RCTs) was conducted across seven databases from their inception to 31 October 2024. The analysis included outcomes such as efficacy rate, overall symptom score, abdominal pain score, diarrhea score, abdominal distension score, loss of appetite score, recurrence rate, and adverse events. Meta-analyses were performed using either a random-effects or fixed-effects model. Trial sequential analysis (TSA) was applied to estimate the sample size and validate the robustness of the meta-analysis.

**Results:**

A total of 34 RCTs involving 2,976 participants met the inclusion criteria. The findings demonstrated that SSW alone (RR = 1.28; 95% CI: 1.21, 1.34; P < 0.00001) or combined with biomedicine (RR = 1.26; 95% CI: 1.18, 1.35; P < 0.00001) significantly improved treatment efficacy compared to biomedicine alone. SSW also reduced the overall symptom score (SMD = −1.06; 95% CI: −1.50, −0.61; Z = 4.66; P < 0.00001) and alleviated key symptoms, including abdominal pain (MD = −0.66; 95% CI: −0.76, −0.56; Z = 12.99; P < 0.00001), diarrhea (MD = −0.69; 95% CI: −0.81, −0.56; Z = 10.82; P < 0.00001), abdominal distension (MD = −0.65; 95% CI: −1.06, −0.24; Z = 3.13; P = 0.002), and loss of appetite (MD = −0.55; 95% CI: −0.66, −0.44; Z = 9.80; P < 0.00001). The recurrence rate was also significantly reduced (RR = 0.40; 95% CI: 0.29, 0.55; P < 0.00001). Additionally, SSW combined with moxibustion—a traditional Chinese medicine therapy integrating internal and external treatments—also further improved treatment outcomes (RR = 1.22; 95% CI: 1.08, 1.37; P = 0.0001). This combination effectively reduced abdominal pain (MD = −0.42; 95% CI: −0.81, −0.04; Z = 2.17; P = 0.03), diarrhea (MD = −0.41; 95% CI: −0.64, −0.17; Z = 3.41; P = 0.0006), abdominal distension (MD = −0.40; 95% CI: −0.69, −0.11; Z = 2.67; P = 0.008), and loss of appetite (MD = −0.30; 95% CI: −0.49, −0.10; Z = 2.93; P = 0.003). Safety analysis revealed a high level of safety for SSW and SSW combined with moxibustion, with no serious adverse events reported in any of the included trials. TSA confirmed an adequate sample size for the primary outcome, supporting the efficacy of SSW in IBS-D treatment.

**Conclusion:**

SSW, either used alone or combined with moxibustion, is effective in alleviating IBS-D symptoms and reducing recurrence rates, making it a potentially beneficial intervention. However, certain limitations remain in the overall quality of the current studies, including relatively small sample sizes, insufficiently long follow-up periods, and the absence of a double-blind design. Future research should emphasize the design and implementation of high-quality, long-term, randomized, double-blind clinical trials to further enhance the reliability and external applicability of the research findings.

**Systematic Review Registration:**

https://www.crd.york.ac.uk/PROSPERO/display_record.php?RecordID=597979, identifier CRD42024597979.

## 1 Introduction

Irritable bowel syndrome (IBS) is one of the prevalent clinical functional gastrointestinal disorders (FGIDs) as defined by the Rome IV criteria (Drossman and Hasler, 2016). It is characterized by recurrent abdominal pain associated with alterations in bowel habits, including changes in stool frequency and consistency. Based on distinct bowel habit patterns, IBS is classified into four subtypes: IBS with predominant constipation (IBS-C), IBS with predominant diarrhea (IBS-D), IBS with mixed bowel habits (IBS-M), and IBS unclassified (IBS-U). Among these, the prevalence of IBS-D is high in clinical practice. As a chronic functional disorder, IBS is notable for its high prevalence, long duration, and recurrent nature. The global prevalence is estimated to be approximately 11.2% (Enck et al., 2016; Sperber et al., 2016). This condition not only significantly diminishes patients’ quality of life but also increases healthcare costs and reduces productivity, imposing a substantial economic burden on society (Zhang et al., 2017; Lovell and Ford, 2012; Nellesen et al., 2013; Agarwal and Spiegel, 2011). Current research indicates that the pathogenesis of IBS is multifactorial, involving brain-gut axis dysregulation, psychological and emotional factors, abnormal intestinal motility, visceral hypersensitivity, gut microbiota dysbiosis, and genetic predisposition. Due to its complex pathogenesis, no specific drug is available for IBS treatment, and most therapies focus on symptom management. Common treatment approaches include pharmacotherapy, dietary interventions, and psychological therapies, but their efficacy remains suboptimal.

Traditional Chinese Medicine (TCM) has demonstrated unique advantages in managing functional disorders, including IBS. A meta-analysis of 49 studies indicated that TCM is both effective and well-tolerated for treating FGIDs (Tan et al., 2020). Si-Shen-Wan (SSW), a classical TCM prescription, is primarily used to treat diarrhea caused by spleen and kidney Yang deficiency and is commonly applied in IBS-D management. Although SSW has been widely used in clinical practice, its specific therapeutic effects on IBS-D remain unclear. Some randomized controlled trials (RCTs) suggest that modifications of SSW yield favorable outcomes for IBS-D. However, high-quality meta-analyses are still lacking. This study aims to perform a meta-analysis to evaluate the efficacy of SSW in treating IBS-D using evidence-based medicine. The meta-analysis incorporates trial sequential analysis (TSA) to objectively assess existing research and provide guidance for future clinical practice and research.

## 2 Methods

### 2.1 Search strategy

This meta-analysis adhered to the Preferred Reporting Items for Systematic Reviews and Meta-analyses (PRISMA) guidelines. A comprehensive search was conducted across seven databases: PubMed, EMBASE, Cochrane Library, China National Knowledge Infrastructure (CNKI), Wanfang, Chinese Scientific Journals Database (VIP), and Chinese Biological Medical Database (CBM). The search period extended from the inception of each database to 31 October 2024. Additionally, dissertations related to clinical trials were retrieved from CNKI and Wanfang. The literature search utilized a combination of subject terms and free-text keywords, including “irritable bowel syndrome,” “Sishen,” and “randomized controlled trials.” Details of the complete search strategy are provided in a Supplementary Document. To ensure comprehensive inclusion, systematic reviews and meta-analyses relevant to IBS were also examined. This meta-analysis has been registered on PROSPERO (Registration Number: CRD42024597979).

### 2.2 Study selection

The inclusion criteria for the study were as follows: 1. The data comprised RCTs published in Chinese or English that explicitly described the randomization method or mentioned the term “randomization.” 2. The study population included adult patients diagnosed with IBS-D based on established diagnostic criteria, irrespective of gender or ethnicity. 3. The experimental group received SSW or TCM based on SSW, combined with biomedicine (the same biomedicine was used in both experimental and control groups within each study). TCM could be administered in various forms, such as decoctions, granules, capsules, tablets, or powders. 4. The control group received conventional drugs for IBS-D without restrictions on drug categories or quantities. 5. The primary outcome measure was the efficacy rate, while secondary outcomes included symptom scores and the incidence of adverse reactions.

The exclusion criteria were: 1. Retracted or duplicate articles. 2. Reviews, case reports, animal studies, or experimental summaries. 3. Retrospective analyses. 4. Studies in which the control group received a different biomedicine treatment from the experimental group. 5. Articles with incomplete case records, insufficient reporting, or non-extractable data.

### 2.3 Data extraction

Two researchers independently reviewed the titles, abstracts, and full texts of the identified literature, applying the predefined inclusion and exclusion criteria. Studies meeting the criteria were included. Extracted data included: Literature title, author information, and publication date. Sample size and diagnostic criteria for IBS-D. Characteristics of the study population and baseline data consistency. Intervention measures, treatment details, and follow-up duration. Outcome evaluation indicators and results. Reported adverse drug reactions. Discrepancies between researchers were resolved through consultation with a third party. For clinical trials with multiple publications reporting different outcomes, the data were consolidated for inclusion.

### 2.4 Methodological quality assessment

The risk of bias in the included studies was assessed using Review Manager 5.3 software provided by the Cochrane Collaboration. The evaluation criteria included the following domains: generation of randomized sequences, allocation concealment, blinding of investigators and participants, blinded assessment of study outcomes, completeness of outcome data, selective reporting of results, and other potential sources of bias. Two researchers independently assessed the methodological quality of the studies, classifying the risk of bias as low, unclear, or high based on these criteria. Any disagreements between the two evaluators were resolved through consultation with a third reviewer.

### 2.5 Statistical analysis

The pooled effect size was analyzed using Review Manager 5.3 (RevMan for Windows, Cochrane Collaboration, Oxford, UK). For dichotomous variables, relative risk (RR) was employed, while mean difference (MD) was used for continuous variables measured in the same units. If continuous variables were measured in different units, the standardized mean difference (SMD) was applied. All results were reported with 95% confidence intervals (CIs). Heterogeneity among the included studies was assessed using the χ2 test and the I^2^ statistic. If the P-value from the χ2 test was greater than 0.10 and the I^2^ statistic was less than 50%, the heterogeneity was considered acceptable, and a fixed-effects model was applied to compute the pooled statistics. Conversely, if significant heterogeneity was detected (P ≤ 0.10 or I^2^ ≥ 50%), a random-effects model was used instead.

### 2.6 Trial sequential analysis

To address the increased risk of random errors caused by sparse data and repeated significance testing, trial sequential analysis (TSA) was performed for the primary outcomes using TSA software version 0.9.5.10 beta. The threshold for a Type I error (α) was set at 0.05, and the threshold for a Type II error (β) was set at 0.2, corresponding to a statistical power of 80%. The required sample size, referred to as the required information size (RIS), was calculated based on the results of the meta-analysis, using the relative risk reduction (RRR) and control group event rate as parameters.

## 3 Results

### 3.1 Study selection

**FIGURE 1:**
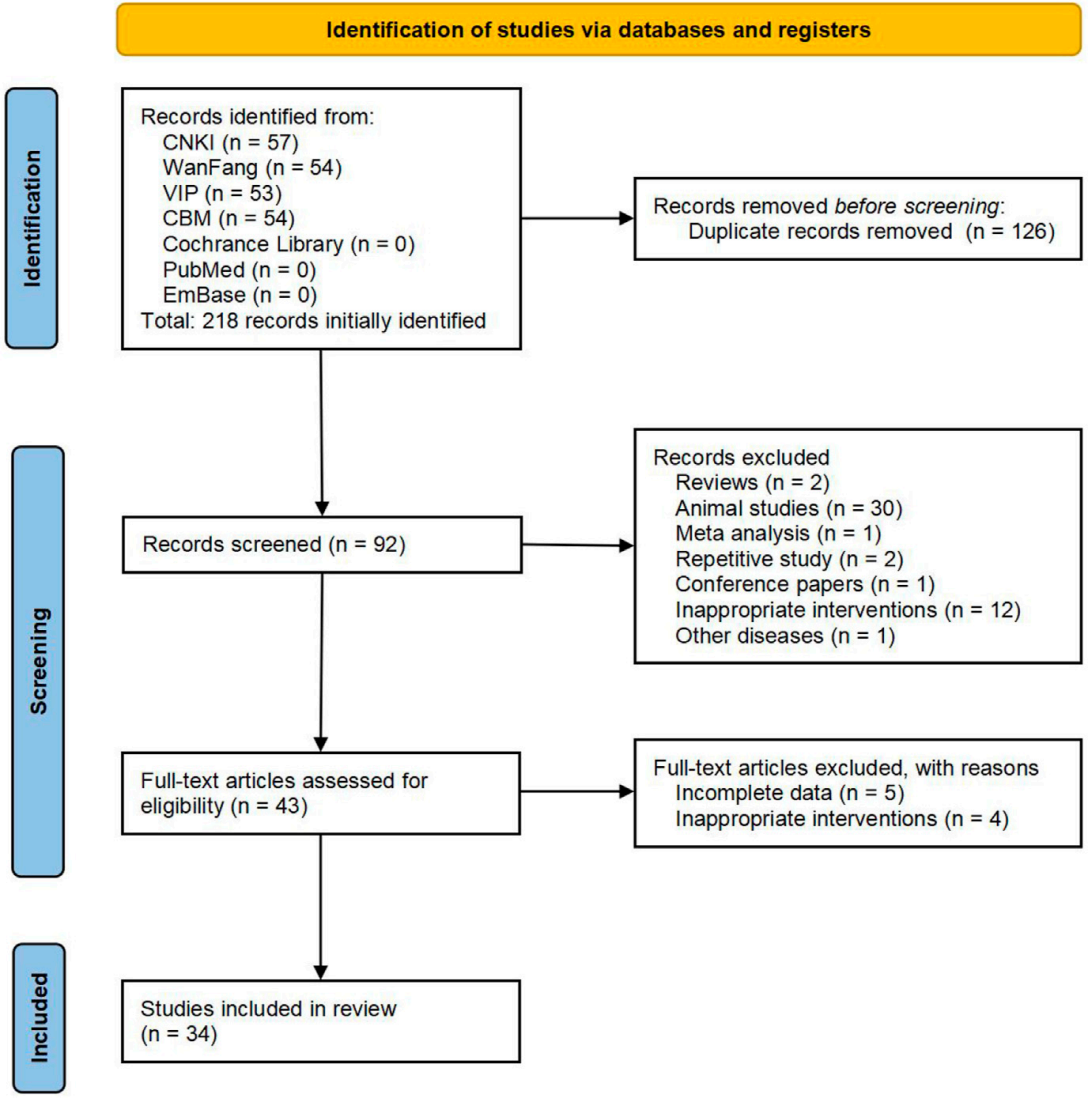
A flowchart of literature search and selection process.

According to the predefined inclusion and exclusion criteria, 218 Chinese articles were identified, while no English articles met the selection criteria. After removing 126 duplicate articles, the abstracts of the remaining 92 articles were reviewed. Of these, 49 were excluded because they were reviews, animal experiments, or studies irrelevant to the search strategy. Full texts of the remaining 43 articles were examined in detail, and studies with incomplete data or inadequate intervention measures were excluded. Ultimately, 34 studies were deemed eligible and included in the meta-analysis. The detailed literature screening process is illustrated in Figure 1.

### 3.2 Characteristics of included studies

A total of 34 articles were included, comprising four doctoral theses (Bian, 2017; Chen, 2021; Sun, 2023; Xiao, 2021) and 30 journal articles (Chen et al., 2018; Chen et al., 2021; Chen, 2017; Cheng, 2010; Fu et al., 2018; Gu et al., 2016; He and Liao, 2017; Hou, 2018; Hu et al., 2023; Hu, 2016; Hu and Zhang, 2020; Huang, 2018; Huang et al., 2016; Li et al., 2022; Li and Li, 2019; Li and Li, 2018; Liang et al., 2020; Liu, 2013; Luo et al., 2019; Nie and Zou, 2014; Wen, 2012; Wen and Cao, 2016; Wu et al., 2023; Xue et al., 2024; Yang and Zhang, 2009; Yang, 2015; Ye, 2010; Zhang et al., 2022; Zhang, 2021; Zhao, 2020), and involving 2,976 patients. Among these, 25 studies used the Rome Committee diagnostic criteria for IBS-D, while other diagnostic criteria were employed in the remaining studies. The treatment group interventions included: 21 studies using SSW as the basic prescription; 10 studies combining SSW with biomedicine. Three studies combining SSW with moxibustion. In all studies, the control group received conventional biomedicine. In all studies, the baseline characteristics (age, sex, course of disease, symptoms) of the participants in various treatment groups were comparable. The basic characteristics of the included studies are summarized in Table 1. The ingredients of SSW used in 34 studies were summarized in Table 2.

**TABLE 1:** Characteristics of the included trials.

Authors (Year)	Diagnostic Criteria	Sample size (T/C)	Age (years) (Mean ± SD) OR Min-Max (Mean)	Disease duration (years) (Mean ± SD) OR Min-Max (Mean)	Intervention		Duration (days)	Outcome measurements	Adverse event (Patients, n)
					T	C			
Bian (2017)	Rome III	30/30	T 34.60 ± 11.19 C 36.03 ± 11.76	T 2.26 ± 1.33 C 2.40 ± 1.43	Modified SSW	Live Combined Bifidobacterium and *Lactobacillus* Tablets (2 g tid)	28	1 Clinical therapeutic efficacy 2 Symptom score	T 0/30 C 0/30
Chen et al. (2018)	Rome III	49/49	T 39.13 ± 6.27 C 38.21 ± 7.54	T 8.89 ± 1.92 C 8.68 ± 1.23	Modified SSW	Trimebutine Maleate Tablets (0.2 g tid) Oryzanol Tablets (30 mg tid)	28	1 Clinical therapeutic efficacy 2 Symptom score	T 1/49 C 8/49
Chen et al. (2021)	Rome IV	44/46	T 49.25 ± 14.5 C 46.57 ± 14.18	T 4.32 ± 3.11 C 5.67 ± 3.93	SSW + moxibustion	Pinaverium Bromide Tablets (50 mg tid)	28	1 Clinical therapeutic efficacy 2 Symptom score	NR
Chen (2021)	Rome IV	30/30	NR	NR	Modified SSW	Montmorillonite powder (3 g bid) Live Combined Bifidobacterium,*Lactobacillus* and *Enterococcus* Capsules (0.63 g bid)	14	1 Symptom score	T 2/30 C 0/30
Chen (2017)	Rome III	45/45	T 20–73 C 21–70	T NR C NR	SSW + C	*Lactobacillus* Complex Capsules (0.66 g tid)	28	1 Symptom score	NR
Cheng (2010)	Rome III	32/32	T 46.2 C 45.2	T 11–108 (M) C 12–90 (M)	Modified SSW	Pinaverium Bromide Tablets (100 mg tid)	84	1 Clinical therapeutic efficacy 2 Symptom score 3 Rate of recurrence	NR
Fu et al. (2018)	Rome III	35/35	T 43.5 ± 8.7 C 44.1 ± 8.6	T 11.6 ± 4.5 C 12.2 ± 4.6	Modified SSW + moxibustion	Pinaverium Bromide Tablets (50 mg tid)	28	1 Clinical therapeutic efficacy 2 Symptom score	T 2/35 C 2/35
Gu et al. (2016)	Rome III	30/30	T 43.5 ± 4.2 C 42.9 ± 3.9	T 5.5 ± 2.3 C 5.7 ± 2.6	Modified SSW	Pinaverium Bromide Tablets (50 mg tid)	30	1 Clinical therapeutic efficacy 2 Symptom score	NR
He and Liao (2017)	Rome III	20/21	T 33.4 ± 8.9 C 30.8 ± 8.7	T 14.6 ± 3.4 C 13.2 ± 4.3	SSW	Loperamide Hydrochloride Capsules (2 mg bid)	28	1 Symptom score	NR
Hou (2018)	Rome III	50/50	T 55.71 ± 6.95 C 55.58 ± 6.90	T 2.30 ± 0.67 C 2.37 ± 0.70	Modified SSW + C	Trimebutine Maleate Tablets (0.2 g tid)	56	1 Clinical therapeutic efficacy 2 Symptom score 3 Rate of recurrence	NR
Hu et al. (2023)	Rome III	64/60	T 33.47 ± 3.14 C 34.56 ± 3.81	T 1.17 ± 0.31 C 1.17 ± 0.24	Modified SSW + C	*Bacillus* coagulans Tablets (0.35 g tid)	28	1 Clinical therapeutic efficacy 2 Symptom score	NR
Hu (2016)	Rome III	55/55	T 45.5 ± 5.3 C 45.8 ± 5.5	T 23.5 ± 5.3 C 23.8 ± 5.5	Modified SSW	Pinaverium Bromide Tablets (50 mg tid)	28	1 Clinical therapeutic efficacy	NR
Hu and Zhang (2020)	Other	50/50	T 59.26 ± 3.17 C 58.7 ± 3.36	NR	Modified SSW	Live Combined *Bacillus Subtilis* and *Enterococcus* Faecium Enteric-coated Capsules (0.5 g bid)	42	1 Symptom score	NR
Huang (2018)	Other	44/43	T 35.63 ± 10.35 C 36.56 ± 10.42	T 5.42 ± 1.07 C 5.3 ± 1.4	Modified SSW + C	Pinaverium Bromide Tablets (50 mg tid)	28	1 Clinical therapeutic efficacy	T 6/44 C 4/43
Huang et al. (2016)	Other	32/30	T 48.6 ± 3.2 C 49.1 ± 4.2	NR	SSW + C	Trimebutine Maleate Tablets (0.2 g tid)	28	1 Clinical therapeutic efficacy 2 Symptom score	NR
Li et al. (2022)	Rome III	47/47	T 38.59 ± 6.37 C 36.84 ± 6.49	T 3.32 ± 0.61 C 3.17 ± 0.58	SSW	Pinaverium Bromide Tablets (50 mg tid)	28	1 Clinical therapeutic efficacy 2 Symptom score	T 0/47 C 0/47
Li and Li (2019)	Rome IV	40/40	T 35 ± 1.8 C 36 ± 1.6	T 6.1 ± 1.28 C 6.1 ± 1.18	Modified SSW	Trimebutine Maleate Tablets (0.1 g tid)	14	1 Symptom score	NR
Li and Li (2018)	Rome III	37/37	T 39.6 ± 3.1 C 39.3 ± 3.5	T 5.8 ± 1.2 C 5.7 ± 1.4	Modified SSW	Pinaverium Bromide Tablets (50 mg tid) Live Combined *Bacillus Subtilis* and *Enterococcus* Faecium Enteric-coated Capsules (0.5 g bid)	28	1 Clinical therapeutic efficacy	NR
Liang et al. (2020)	Other	62/62	T 48.47 ± 5.79 C 48.46 ± 5.78	T 3.25 ± 1.04 C 3.23 ± 1.03	SSW + C	Pinaverium Bromide Tablets (50 mg tid) *Clostridium* butyricum Tablets (700 mg tid)	14	1 Symptom score	T 17/62 C 19/62
Liu (2013)	Rome III	76/72	T 44.2 ± 3.8 C 43.8 ± 3.6	T 3.4 ± 1.5 C 3.3 ± 1.4	Modified SSW	Trimebutine Maleate Tablets (0.1 g tid)	28	1 Clinical therapeutic efficacy 2 Rate of recurrence	T 0/76 C 0/72
Luo et al. (2019)	Rome IV	30/30	T 36.52 ± 7.21 C 37.13 ± 7.58	T 3.85 ± 0.89 C 3.65 ± 0.76	Modified SSW + C	Biomedicine	12	1 Clinical therapeutic efficacy 2 Rate of recurrence	NR
Nie and Zou (2014)	Rome III	30/30	T 52.20 ± 8.29 C 51.64 ± 7.69	T 57.48 ± 36.84 (M) C 59.08 ± 34.08 (M)	SSW	Montmorillonite powder (3 g bid)	14	1 Clinical therapeutic efficacy 2 Symptom score	T 0/30 C 0/30
Sun (2023)	Rome IV	36/36	T 40.11 ± 12.54 C 38.83 ± 12.18	T 26.34 ± 14.32 (M) C 24.29 ± 11.59 (M)	Modified SSW	Trimebutine Maleate Tablets (0.2 g tid) Compound Eosinophil-Lactobacillus Tablets (1 g tid)	56	1 Clinical therapeutic efficacy 2 Symptom score 3 Rate of recurrence	T 0/36 C 0/36
Wen (2012)	Rome III	40/40	NR	NR	Modified SSW	Pinaverium Bromide Tablets (100 mg tid) Live Combined Bifidobacterium,*Lactobacillus* and *Enterococcus* Capsules (0.42 g tid)	28	1 Clinical therapeutic efficacy 2 Rate of recurrence	NR
Wen and Cao (2016)	Rome III	60/60	T 57.82 ± 6.23 C 56.49 ± 6.17	T 2.26 ± 0.25 C 2.19 ± 0.23	modified SSW	Live Combined *Bacillus Subtilis* and *Enterococcus* Faecium Enteric-coated Capsules (0.5 g bid)	42	1 Clinical therapeutic efficacy 2 Symptom score	NR
Wu et al. (2023)	Rome III	40/40	T 47.02 ± 2.16 C 46.98 ± 2.12	T 3.25 ± 1.04 C 3.62 ± 0.33	Modified SSW + C	Trimebutine Maleate Tablets (0.2 g tid) Loperamide Hydrochloride Capsules (2 mg bid)	28	1 Clinical therapeutic efficacy 2 Symptom score 3 Rate of recurrence	NR
Xiao (2021)	Rome IV	30/29	T 39.37 ± 9.67 C 40.51 ± 9.95	T 18.87 ± 4.46 (M) C 20.38 ± 5.17 (M)	Modified SSW	Pinaverium Bromide Tablets (50 mg tid)	28	1 Clinical therapeutic efficacy 2 Symptom score 3 Rate of recurrence	NR
Xue et al. (2024)	Other	53/53	T 56.20 ± 6.18 C 56.47 ± 6.27	T 9.62 ± 1.17 (M) C 9.45 ± 1.21 (M)	Modified SSW + C	Pinaverium Bromide Tablets (50 mg tid)	28	1 Symptom score	T 5/53 C 3/53
Yang and Zhang (2009)	Other	54/32	T 37.4 C 39.8	T 8.5 C 8.5	Modified SSW	Live Combined Bifidobacterium and *Lactobacillus* Tablets (2 g tid)	28	1 Clinical therapeutic efficacy 2 Rate of recurrence	NR
Yang (2015)	Other	42/42	43.2 ± 1.6	22 ± 1.3	Modified SSW	Pinaverium Bromide Tablets (50 mg tid)	42	1 Clinical therapeutic efficacy	NR
Ye (2010)	Rome II	62/33	T 38.3 C 39.8	T 5.5 C 5	Modified SSW	Montmorillonite powder (3 g tid)	56	1 Clinical therapeutic efficacy	T 0/62 C 0/33
Zhang et al. (2022)	Rome III	40/40	T 51.95 ± 9.83 C 52.15 ± 9.56	T 8.92 ± 2.6 (M) C 9.05 ± 2.35 (M)	SSW + moxibustion	Montmorillonite powder (3 g tid) *Bacillus* coagulans Tablets (0.35 g tid)	20	1 Clinical therapeutic efficacy 2 Symptom score	NR
Zhang (2021)	Other	48/48	T 49.46 ± 10.08 C 48.26 ± 9.42	T 2.58 ± 0.98 C 2.82 ± 1.04	Modified SSW	Trimebutine Maleate Tablets (0.1 g tid)	28	1 Clinical therapeutic efficacy	NR
Zhao (2020)	Other	81/81	T 38.56 ± 3.84 C 38.48 ± 3.81	T 1.61 ± 0.96 C 1.65 ± 0.85	Modified SSW + C	Pinaverium Bromide Tablets (50 mg tid) *Clostridium* butyricum Tablets (700 mg tid)	28	1 Clinical therapeutic efficacy 2 Symptom score	T 6/81 C 3/81

Abbreviations: T, treatment group; C, control group; M, month; NR, not reported.

**TABLE 2:** The ingredients of Si Shen Wan used in the included trials.

Authors (Year)	Ingredients of SSW
Bian (2017)	Myristica fragrans Houtt [Myristicaceae; Myristicae semen] 15g, Psoralea corylifolia [Fabaceae; Psoraleae fructus] 20g, Schisandra chinensis [Schisandraceae; Schisandrae fructus] 15g, Tetradium ruticarpum [Rutaceae; Tetradii fructus] 15g, Zingiber officinale [Zingiberaceae; Zingiberis rhizoma] 6g, Ziziphus jujuba [Rhamnaceae; Ziziphi fructus] 6g, Glycyrrhiza uralensis [Fabaceae; Glycyrrhizae radix et rhizoma] 6g, Codonopsis pilosula [Campanulaceae; Codonopsis radix] 20g, Atractylodes macrocephala [Asteraceae; Atractylodis rhizoma] 15g, Dioscorea opposita [Dioscoreaceae; Dioscoreae rhizoma] 20g, Wolfiporia cocos [Polyporaceae; Poriae sclerotium] 20g, Amomum villosum [Zingiberaceae; Amomi fructus] 5g, Lablab purpureus [Fabaceae; Lablab semen] 10g
Chen et al. (2018)	Myristica fragrans Houtt [Myristicaceae; Myristicae semen] 10g, Psoralea corylifolia [Fabaceae; Psoraleae fructus] 10g, Schisandra chinensis [Schisandraceae; Schisandrae fructus] 10g, Tetradium ruticarpum [Rutaceae; Tetradii fructus] 6g, Citrus reticulata [Rutaceae; Citri reticulatae pericarpium] 10g, Saposhnikovia divaricata [Apiaceae; Saposhnikoviae radix] 10g, Atractylodes macrocephala [Asteraceae; Atractylodis rhizoma] 15g, Codonopsis pilosula [Campanulaceae; Codonopsis radix] 15g, Wolfiporia cocos [Polyporaceae; Poriae sclerotium] 15g, Paeonia lactiflora [Paeoniaceae; Paeoniae radix] 20g
Chen et al. (2021)	Sishen Pills (Z13021159, Yaodu Pharmaceutical Group Co., Ltd.)
Chen (2021)	Myristica fragrans Houtt [Myristicaceae; Myristicae semen] 10g, Psoralea corylifolia [Fabaceae; Psoraleae fructus] 15g, Schisandra chinensis [Schisandraceae; Schisandrae fructus] 6g, Tetradium ruticarpum [Rutaceae; Tetradii fructus] 3g, Zingiber officinale [Zingiberaceae; Zingiberis rhizoma] 6g, Bupleurum chinense [Apiaceae; Bupleuri radix] 12g, Paeonia lactiflora [Paeoniaceae; Paeoniae radix] 15g, Wolfiporia cocos [Polyporaceae; Poriae sclerotium] 15g, Atractylodes macrocephala [Asteraceae; Atractylodis rhizoma] 24g, Curcuma aromatica [Zingiberaceae; Curcumae rhizoma]12g, Angelica sinensis [Apiaceae; Angelicae radix] 9g, Glycyrrhiza uralensis [Fabaceae; Glycyrrhizae radix et rhizoma] 6g
Chen (2017)	Myristica fragrans Houtt [Myristicaceae; Myristicae semen] 12g, Psoralea corylifolia [Fabaceae; Psoraleae fructus] 20g, Schisandra chinensis [Schisandraceae; Schisandrae fructus] 10g, Tetradium ruticarpum [Rutaceae; Tetradii fructus] 5g, Zingiber officinale [Zingiberaceae; Zingiberis rhizoma] 6g, Ziziphus jujuba [Rhamnaceae; Ziziphi fructus] 10g, Terminalia chebula [Combretaceae; Terminaliae fructus] 8g
Cheng (2010)	Myristica fragrans Houtt [Myristicaceae; Myristicae semen] 10g, Psoralea corylifolia [Fabaceae; Psoraleae fructus] 15g, Schisandra chinensis [Schisandraceae; Schisandrae fructus] 6g, Tetradium ruticarpum [Rutaceae; Tetradii fructus] 6g, Zingiber officinale [Zingiberaceae; Zingiberis rhizoma] 10g, Ziziphus jujuba [Rhamnaceae; Ziziphi fructus] 20g
Fu et al. (2018)	Myristica fragrans Houtt [Myristicaceae; Myristicae semen] 12g, Psoralea corylifolia [Fabaceae; Psoraleae fructus] 12g, Schisandra chinensis [Schisandraceae; Schisandrae fructus] 10g, Tetradium ruticarpum [Rutaceae; Tetradii fructus] 12g, Codonopsis pilosula [Campanulaceae; Codonopsis radix] 12g, Atractylodes macrocephala [Asteraceae; Atractylodis rhizoma] 15g, Dioscorea opposita [Dioscoreaceae; Dioscoreae rhizoma] 15g, Paeonia lactiflora [Paeoniaceae; Paeoniae radix] 10g, Lablab purpureus [Fabaceae; Lablab semen] 15g, Citrus reticulata [Rutaceae; Citri reticulatae pericarpium] 10g, Aucklandia lappa [Asteraceae; Aucklandiae radix] 10g, Cimicifuga foetida [Ranunculaceae; Cimicifugae rhizoma] 10g, Glycyrrhiza uralensis [Fabaceae; Glycyrrhizae radix et rhizoma] 5g
Gu et al. (2016)	Myristica fragrans Houtt [Myristicaceae; Myristicae semen] 10g, Psoralea corylifolia [Fabaceae; Psoraleae fructus] 20g, Schisandra chinensis [Schisandraceae; Schisandrae fructus] 10g, Tetradium ruticarpum [Rutaceae; Tetradii fructus] 9g, Zingiber officinale [Zingiberaceae; Zingiberis rhizoma] 20g, Ziziphus jujuba [Rhamnaceae; Ziziphi fructus] 9g, Punica granatum [Lythraceae; Granati pericarpium] 15g, Codonopsis pilosula [Campanulaceae; Codonopsis radix] 15g, Atractylodes macrocephala [Asteraceae; Atractylodis rhizoma] 15g, Aucklandia lappa [Asteraceae; Aucklandiae radix] 15g, Glycyrrhiza uralensis [Fabaceae; Glycyrrhizae radix et rhizoma] 10g
He and Liao (2017)	Myristica fragrans Houtt [Myristicaceae; Myristicae semen] 6g, Psoralea corylifolia [Fabaceae; Psoraleae fructus] 12g, Schisandra chinensis [Schisandraceae; Schisandrae fructus] 9g, Tetradium ruticarpum [Rutaceae; Tetradii fructus] 6g, Zingiber officinale [Zingiberaceae; Zingiberis rhizoma] 6g, Ziziphus jujuba [Rhamnaceae; Ziziphi fructus] 6=g
Hou (2018)	Myristica fragrans Houtt [Myristicaceae; Myristicae semen] 10g, Psoralea corylifolia [Fabaceae; Psoraleae fructus] 15g, Schisandra chinensis [Schisandraceae; Schisandrae fructus] 15g, Tetradium ruticarpum [Rutaceae; Tetradii fructus] 10g, Zingiber officinale [Zingiberaceae; Zingiberis rhizoma] 10g, Ziziphus jujuba [Rhamnaceae; Ziziphi fructus] 9g, Codonopsis pilosula [Campanulaceae; Codonopsis radix] 20g, Dioscorea opposita [Dioscoreaceae; Dioscoreae rhizoma] 20g, Atractylodes macrocephala [Asteraceae; Atractylodis rhizoma] 15g, Wolfiporia cocos [Polyporaceae; Poriae sclerotium] 15g, Aconitum carmichaelii [Ranunculaceae; Aconiti radix lateralis preparata] 10g
Hu et al. (2023)	Myristica fragrans Houtt [Myristicaceae; Myristicae semen] 15g, Psoralea corylifolia [Fabaceae; Psoraleae fructus] 15g, Schisandra chinensis [Schisandraceae; Schisandrae fructus] 10g, Tetradium ruticarpum [Rutaceae; Tetradii fructus] 5g, Zingiber officinale [Zingiberaceae; Zingiberis rhizoma] 5g, Ziziphus jujuba [Rhamnaceae; Ziziphi fructus] 10g, Codonopsis pilosula [Campanulaceae; Codonopsis radix] 15g, Atractylodes macrocephala [Asteraceae; Atractylodis rhizoma] 15g, Aconitum carmichaelii [Ranunculaceae; Aconiti radix lateralis preparata] 10g, Glycyrrhiza uralensis [Fabaceae; Glycyrrhizae radix et rhizoma] 5g, Wolfiporia cocos [Polyporaceae; Poriae sclerotium] 15g
Hu (2016)	Sishen Pills (Z13020141, Hebei Wansui Pharmaceutical Co., Ltd.)
Hu and Zhang (2020)	Myristica fragrans Houtt [Myristicaceae; Myristicae semen] 12g, Psoralea corylifolia [Fabaceae; Psoraleae fructus] 10g, Schisandra chinensis [Schisandraceae; Schisandrae fructus] 15g, Tetradium ruticarpum [Rutaceae; Tetradii fructus] 9g, Dioscorea opposita [Dioscoreaceae; Dioscoreae rhizoma] 15g, Codonopsis pilosula [Campanulaceae; Codonopsis radix] 15g, Wolfiporia cocos [Polyporaceae; Poriae sclerotium] 10g, Atractylodes macrocephala [Asteraceae; Atractylodis rhizoma] 10g
Huang (2018)	Myristica fragrans Houtt [Myristicaceae; Myristicae semen] 9g, Psoralea corylifolia [Fabaceae; Psoraleae fructus] 9g, Schisandra chinensis [Schisandraceae; Schisandrae fructus] 9g, Tetradium ruticarpum [Rutaceae; Tetradii fructus] 6g, Zingiber officinale [Zingiberaceae; Zingiberis rhizoma] 9g, Ziziphus jujuba [Rhamnaceae; Ziziphi fructus] 9g, Panax ginseng [Araliaceae; Ginseng radix et rhizoma] 9g, Paeonia lactiflora [Paeoniaceae; Paeoniae radix] 9g, Glycyrrhiza uralensis [Fabaceae; Glycyrrhizae radix et rhizoma] 9g, Bupleurum chinense [Apiaceae; Bupleuri radix] 9g, Citrus aurantium [Rutaceae; Aurantii fructus immaturus] 9g
Huang et al. (2016)	Myristica fragrans Houtt [Myristicaceae; Myristicae semen] 6g, Psoralea corylifolia [Fabaceae; Psoraleae fructus] 12g, Schisandra chinensis [Schisandraceae; Schisandrae fructus] 6g, Tetradium ruticarpum [Rutaceae; Tetradii fructus] 3g, Zingiber officinale [Zingiberaceae; Zingiberis rhizoma] 12g, Ziziphus jujuba [Rhamnaceae; Ziziphi fructus] 10g
Li et al. (2022)	Myristica fragrans Houtt [Myristicaceae; Myristicae semen] 12g, Psoralea corylifolia [Fabaceae; Psoraleae fructus] 10g, Schisandra chinensis [Schisandraceae; Schisandrae fructus] 15g, Tetradium ruticarpum [Rutaceae; Tetradii fructus] 9g, Zingiber officinale [Zingiberaceae; Zingiberis rhizoma] 6g, Ziziphus jujuba [Rhamnaceae; Ziziphi fructus] 9g
Li and Li (2019)	Myristica fragrans Houtt [Myristicaceae; Myristicae semen] 15g, Psoralea corylifolia [Fabaceae; Psoraleae fructus] 20g, Schisandra chinensis [Schisandraceae; Schisandrae fructus] 15g, Tetradium ruticarpum [Rutaceae; Tetradii fructus] 20g, Ziziphus jujuba [Rhamnaceae; Ziziphi fructus] 10g, Panax ginseng [Araliaceae; Ginseng radix et rhizoma] 10g, Atractylodes macrocephala [Asteraceae; Atractylodis rhizoma] 35g, Wolfiporia cocos [Polyporaceae; Poriae sclerotium] 10g, Glycyrrhiza uralensis [Fabaceae; Glycyrrhizae radix et rhizoma] 10g, Aucklandia lappa [Asteraceae; Aucklandiae radix] 10g, Coptis chinensis [Ranunculaceae; Coptidis rhizoma] 10g, Amomum villosum [Zingiberaceae; Amomi fructus] 10g, Dioscorea opposita [Dioscoreaceae; Dioscoreae rhizoma] 30g, Massa medicata fermentata 20g, Punica granatum [Lythraceae; Granati pericarpium] 30g, Halloysitum rubrum 20g, Plantago asiatica [Plantaginaceae; Plantaginis semen] 20g
Li and Li (2018)	Myristica fragrans Houtt [Myristicaceae; Myristicae semen] 25g, Psoralea corylifolia [Fabaceae; Psoraleae fructus] 30g, Schisandra chinensis [Schisandraceae; Schisandrae fructus] 10g, Tetradium ruticarpum [Rutaceae; Tetradii fructus] 9g, Zingiber officinale [Zingiberaceae; Zingiberis rhizoma] 7g, Atractylodes macrocephala [Asteraceae; Atractylodis rhizoma] 15g, Wolfiporia cocos [Polyporaceae; Poriae sclerotium] 15g, Codonopsis pilosula [Campanulaceae; Codonopsis radix] 15g, Punica granatum [Lythraceae; Granati pericarpium] 9g, Glycyrrhiza uralensis [Fabaceae; Glycyrrhizae radix et rhizoma] 10g
Liang et al. (2020)	Myristica fragrans Houtt [Myristicaceae; Myristicae semen] 10g, Psoralea corylifolia [Fabaceae; Psoraleae fructus] 10g, Schisandra chinensis [Schisandraceae; Schisandrae fructus] 10g, Tetradium ruticarpum [Rutaceae; Tetradii fructus] 6g, Zingiber officinale [Zingiberaceae; Zingiberis rhizoma] 9g, Ziziphus jujuba [Rhamnaceae; Ziziphi fructus] 9g, Citrus reticulata [Rutaceae; Citri reticulatae pericarpium] 10g, Saposhnikovia divaricata [Apiaceae; Saposhnikoviae radix] 10g, Atractylodes macrocephala [Asteraceae; Atractylodis rhizoma] 15g, Codonopsis pilosula [Campanulaceae; Codonopsis radix] 15g, Wolfiporia cocos [Polyporaceae; Poriae sclerotium] 15g, Paeonia lactiflora [Paeoniaceae; Paeoniae radix] 20g
Liu (2013)	Myristica fragrans Houtt [Myristicaceae; Myristicae semen] 15g, Psoralea corylifolia [Fabaceae; Psoraleae fructus] 15g, Schisandra chinensis [Schisandraceae; Schisandrae fructus] 9g, Tetradium ruticarpum [Rutaceae; Tetradii fructus] 3g, Zingiber officinale [Zingiberaceae; Zingiberis rhizoma] 9g, Citrus aurantium [Rutaceae; Aurantii fructus] 12g, Paeonia lactiflora [Paeoniaceae; Paeoniae radix] 15g, Bupleurum chinense [Apiaceae; Bupleuri radix] 15g, Glycyrrhiza uralensis [Fabaceae; Glycyrrhizae radix et rhizoma] 6g, Wolfiporia cocos [Polyporaceae; Poriae sclerotium] 20g, Saposhnikovia divaricata [Apiaceae; Saposhnikoviae radix] 12g, Terra Flava Usta 30g, Coptis chinensis [Ranunculaceae; Coptidis rhizoma] 6 g
Luo et al. (2019)	Myristica fragrans Houtt [Myristicaceae; Myristicae semen] 15g, Psoralea corylifolia [Fabaceae; Psoraleae fructus] 15g, Schisandra chinensis [Schisandraceae; Schisandrae fructus] 10g, Tetradium ruticarpum [Rutaceae; Tetradii fructus] 6g, Zingiber officinale [Zingiberaceae; Zingiberis rhizoma] 6g, Ziziphus jujuba [Rhamnaceae; Ziziphi fructus] 15g, Codonopsis pilosula [Campanulaceae; Codonopsis radix] 15g, Atractylodes macrocephala [Asteraceae; Atractylodis rhizoma] 12g, Glycyrrhiza uralensis [Fabaceae; Glycyrrhizae radix et rhizoma] 6g, Aconitum carmichaelii [Ranunculaceae; Aconiti radix lateralis preparata] 10g, Wolfiporia cocos [Polyporaceae; Poriae sclerotium] 15g
Nie and Zou (2014)	Myristica fragrans Houtt [Myristicaceae; Myristicae semen] 15g, Psoralea corylifolia [Fabaceae; Psoraleae fructus] 20g, Schisandra chinensis [Schisandraceae; Schisandrae fructus] 12g, Tetradium ruticarpum [Rutaceae; Tetradii fructus] 12g, Zingiber officinale [Zingiberaceae; Zingiberis rhizoma] 9g, Ziziphus jujuba [Rhamnaceae; Ziziphi fructus] 9g
Sun (2023)	Myristica fragrans Houtt [Myristicaceae; Myristicae semen] 10g, Psoralea corylifolia [Fabaceae; Psoraleae fructus] 10g, Schisandra chinensis [Schisandraceae; Schisandrae fructus] 9g, Tetradium ruticarpum [Rutaceae; Tetradii fructus] 3g, Zingiber officinale [Zingiberaceae; Zingiberis rhizoma] 9g, Ziziphus jujuba [Rhamnaceae; Ziziphi fructus] 9g, Aconitum carmichaelii [Ranunculaceae; Aconiti radix lateralis preparata] 9g, Codonopsis pilosula [Campanulaceae; Codonopsis radix] 15g, Wolfiporia cocos [Polyporaceae; Poriae sclerotium] 20g, Atractylodes macrocephala [Asteraceae; Atractylodis rhizoma] 15g, Dioscorea opposita [Dioscoreaceae; Dioscoreae rhizoma] 15g, Glycyrrhiza uralensis [Fabaceae; Glycyrrhizae radix et rhizoma] 9g
Wen (2012)	Myristica fragrans Houtt [Myristicaceae; Myristicae semen] 10g, Psoralea corylifolia [Fabaceae; Psoraleae fructus] 15g, Schisandra chinensis [Schisandraceae; Schisandrae fructus] 6g, Tetradium ruticarpum [Rutaceae; Tetradii fructus] 6g, Zingiber officinale [Zingiberaceae; Zingiberis rhizoma] 9g, Ziziphus jujuba [Rhamnaceae; Ziziphi fructus] 15g, Codonopsis pilosula [Campanulaceae; Codonopsis radix] 15g, *Nelumbo nucifera* [Nelumbonaceae; Nelumbinis semen] 15g, Wolfiporia cocos [Polyporaceae; Poriae sclerotium] 15g, Atractylodes macrocephala [Asteraceae; Atractylodis rhizoma] 15g, Dioscorea opposita [Dioscoreaceae; Dioscoreae rhizoma] 15g, Citrus reticulata [Rutaceae; Citri reticulatae pericarpium] 6g, Platycodon grandiflorus [Campanulaceae; Platycodonis radix] 6g, Amomum villosum [Zingiberaceae; Amomi fructus] 6g
Wen and Cao (2016)	Myristica fragrans Houtt [Myristicaceae; Myristicae semen] 12g, Psoralea corylifolia [Fabaceae; Psoraleae fructus] 10g, Schisandra chinensis [Schisandraceae; Schisandrae fructus] 15g, Tetradium ruticarpum [Rutaceae; Tetradii fructus] 9g, Codonopsis pilosula [Campanulaceae; Codonopsis radix] 15g, Atractylodes macrocephala [Asteraceae; Atractylodis rhizoma] 10g, Wolfiporia cocos [Polyporaceae; Poriae sclerotium] 10g, Dioscorea opposita [Dioscoreaceae; Dioscoreae rhizoma] 15g
Wu et al. (2023)	Myristica fragrans Houtt [Myristicaceae; Myristicae semen] 9g, Psoralea corylifolia [Fabaceae; Psoraleae fructus] 9g, Schisandra chinensis [Schisandraceae; Schisandrae fructus] 9g, Tetradium ruticarpum [Rutaceae; Tetradii fructus] 9g, Zingiber officinale [Zingiberaceae; Zingiberis rhizoma] 9g, Ziziphus jujuba [Rhamnaceae; Ziziphi fructus] 9g, Aconitum carmichaelii [Ranunculaceae; Aconiti radix lateralis preparata] 10g, Atractylodes macrocephala [Asteraceae; Atractylodis rhizoma] 15g, Wolfiporia cocos [Polyporaceae; Poriae sclerotium] 15g, Panax ginseng [Araliaceae; Ginseng radix et rhizoma] 15g, Zingiber officinale [Zingiberaceae; Zingiberis rhizoma] 15g, Glycyrrhiza uralensis [Fabaceae; Glycyrrhizae radix et rhizoma] 15g
Xiao (2021)	Myristica fragrans Houtt [Myristicaceae; Myristicae semen] 15g, Psoralea corylifolia [Fabaceae; Psoraleae fructus] 10g, Schisandra chinensis [Schisandraceae; Schisandrae fructus] 10g, Tetradium ruticarpum [Rutaceae; Tetradii fructus] 5g, Zingiber officinale [Zingiberaceae; Zingiberis rhizoma] 8g, Aconitum carmichaelii [Ranunculaceae; Aconiti radix lateralis preparata] 8g, Codonopsis pilosula [Campanulaceae; Codonopsis radix] 15g, Atractylodes macrocephala [Asteraceae; Atractylodis rhizoma] 10g, Glycyrrhiza uralensis [Fabaceae; Glycyrrhizae radix et rhizoma] 6g, Alpinia oxyphylla [Zingiberaceae; Alpiniae fructus] 10g
Xue et al. (2024)	Sishen Pills (Z14021177, Shanxi Kangwei Pharmaceutical Co., Ltd.)
Yang and Zhang (2009)	Myristica fragrans Houtt [Myristicaceae; Myristicae semen] 6g, Psoralea corylifolia [Fabaceae; Psoraleae fructus] 10g, Schisandra chinensis [Schisandraceae; Schisandrae fructus] 9g, Tetradium ruticarpum [Rutaceae; Tetradii fructus] 6g, Zingiber officinale [Zingiberaceae; Zingiberis rhizoma] 6g, Ziziphus jujuba [Rhamnaceae; Ziziphi fructus] 6g
Yang (2015)	Myristica fragrans Houtt [Myristicaceae; Myristicae semen] 6g, Psoralea corylifolia [Fabaceae; Psoraleae fructus] 10g, Schisandra chinensis [Schisandraceae; Schisandrae fructus] 6g, Tetradium ruticarpum [Rutaceae; Tetradii fructus] 6g, Zingiber officinale [Zingiberaceae; Zingiberis rhizoma] 9g, Ziziphus jujuba [Rhamnaceae; Ziziphi fructus] 9g, Codonopsis pilosula [Campanulaceae; Codonopsis radix] 15g, Coix lacryma-jobi [Poaceae; Coicis semen] 15g, Dioscorea opposita [Dioscoreaceae; Dioscoreae rhizoma] 15g, Wolfiporia cocos [Polyporaceae; Poriae sclerotium] 15g, Atractylodes macrocephala [Asteraceae; Atractylodis rhizoma] 20g, Amomum villosum [Zingiberaceae; Amomi fructus] 5g, Glycyrrhiza uralensis [Fabaceae; Glycyrrhizae radix et rhizoma] 5g
Ye (2010)	Myristica fragrans Houtt [Myristicaceae; Myristicae semen] 9g, Psoralea corylifolia [Fabaceae; Psoraleae fructus] 12g, Schisandra chinensis [Schisandraceae; Schisandrae fructus] 9g, Tetradium ruticarpum [Rutaceae; Tetradii fructus] 6g, Zingiber officinale [Zingiberaceae; Zingiberis rhizoma] 6g, Ziziphus jujuba [Rhamnaceae; Ziziphi fructus] 6g, Atractylodes macrocephala [Asteraceae; Atractylodis rhizoma] 9g, Aconitum carmichaelii [Ranunculaceae; Aconiti radix lateralis preparata] 6g, Panax ginseng [Araliaceae; Ginseng radix et rhizoma] 6g
Zhang et al. (2022)	Myristica fragrans Houtt [Myristicaceae; Myristicae semen] 10g, Psoralea corylifolia [Fabaceae; Psoraleae fructus] 10g, Schisandra chinensis [Schisandraceae; Schisandrae fructus] 6g, Tetradium ruticarpum [Rutaceae; Tetradii fructus] 6g, Zingiber officinale [Zingiberaceae; Zingiberis rhizoma] 9g, Ziziphus jujuba [Rhamnaceae; Ziziphi fructus] 9g, Lablab purpureus [Fabaceae; Lablab semen] 30g, Codonopsis pilosula [Campanulaceae; Codonopsis radix] 30g, Wolfiporia cocos [Polyporaceae; Poriae sclerotium] 30g, Atractylodes macrocephala [Asteraceae; Atractylodis rhizoma] 10g, Prunus mume [Rosaceae; Mume fructus] 10g
Zhang (2021)	Myristica fragrans Houtt [Myristicaceae; Myristicae semen] 10g, Psoralea corylifolia [Fabaceae; Psoraleae fructus] 15g, Schisandra chinensis [Schisandraceae; Schisandrae fructus] 6g, Tetradium ruticarpum [Rutaceae; Tetradii fructus] 6g, Zingiber officinale [Zingiberaceae; Zingiberis rhizoma] 9g, Ziziphus jujuba [Rhamnaceae; Ziziphi fructus] 20g, Citrus reticulata [Rutaceae; Citri reticulatae pericarpium] 10g, Atractylodes macrocephala [Asteraceae; Atractylodis rhizoma] 10g, Wolfiporia cocos [Polyporaceae; Poriae sclerotium] 20g, Ligusticum chuanxiong [Apiaceae; Ligustici rhizoma] 10g, Paeonia veitchii [Paeoniaceae; Paeoniae radix] 10g, Paeonia lactiflora [Paeoniaceae; Paeoniae radix] 10g, Cyperus rotundus [Cyperaceae; Cyperi rhizoma] 6g, Citrus aurantium [Rutaceae; Aurantii fructus] 10g, Glycyrrhiza uralensis [Fabaceae; Glycyrrhizae radix et rhizoma] 6g
Zhao (2020)	Myristica fragrans Houtt [Myristicaceae; Myristicae semen] 10g, Psoralea corylifolia [Fabaceae; Psoraleae fructus] 20g, Schisandra chinensis [Schisandraceae; Schisandrae fructus] 10g, Tetradium ruticarpum [Rutaceae; Tetradii fructus] 9g, Zingiber officinale [Zingiberaceae; Zingiberis rhizoma] 20g, Ziziphus jujuba [Rhamnaceae; Ziziphi fructus] 9g, Codonopsis pilosula [Campanulaceae; Codonopsis radix] 15g, Atractylodes macrocephala [Asteraceae; Atractylodis rhizoma] 15g, Punica granatum [Lythraceae; Granati pericarpium] 15g, Aucklandia lappa [Asteraceae; Aucklandiae radix] 15g, Glycyrrhiza uralensis [Fabaceae; Glycyrrhizae radix et rhizoma] 10g

### 3.3 Risk of bias assessment

All 34 trials reported randomization. Of these, 20 used random number methods and were assessed as having a low risk of bias, while the remaining 14 did not describe the randomization methods and were assessed as having an unclear risk. None of the studies provided details on allocation concealment, and thus all were evaluated as unclear in this aspect. Similarly, none of the studies described blinding of participants or practitioners, resulting in an unclear risk assessment for blinding. Detailed results of the risk of bias assessment for each study are provided in Table 3.

**TABLE 3:** Assessment of the risk of bias of each included trials.

Authors (Year)	Random sequence generation (selection bias)	Allocation concealment (selection bias)	Blinding of participants and personnel (performance bias)	Blinding of outcome assessment (detection bias)	Incomplete outcome data (attrition bias)	Selective reporting (reporting bias)	Other Bias	Overall risk of bias
Bian (2017)	Unclear	Unclear	Unclear	Unclear	L	L	L	H
Chen et al. (2018)	L	Unclear	Unclear	Unclear	L	L	L	L
Chen et al. (2021)	L	Unclear	Unclear	Unclear	L	L	Unclear	L
Chen (2021)	Unclear	Unclear	Unclear	Unclear	H	L	L	H
Chen (2017)	Unclear	Unclear	Unclear	Unclear	H	L	Unclear	H
Cheng (2010)	Unclear	Unclear	Unclear	Unclear	L	L	Unclear	H
Fu et al. (2018)	L	Unclear	Unclear	Unclear	L	L	L	L
Gu et al. (2016)	L	Unclear	Unclear	Unclear	L	L	Unclear	L
He and Liao (2017)	L	Unclear	Unclear	Unclear	L	L	Unclear	L
Hou (2018)	Unclear	Unclear	Unclear	Unclear	L	L	Unclear	H
Hu et al. (2023)	L	Unclear	Unclear	Unclear	L	L	Unclear	L
Hu (2016)	L	Unclear	Unclear	Unclear	L	L	Unclear	L
Hu and Zhang (2020)	L	Unclear	Unclear	Unclear	H	L	Unclear	H
Huang (2018)	L	Unclear	Unclear	Unclear	L	L	L	L
Huang et al. (2016)	Unclear	Unclear	Unclear	Unclear	H	L	Unclear	H
Li et al. (2022)	L	Unclear	Unclear	Unclear	L	L	L	L
Li and Li (2019)	Unclear	Unclear	Unclear	Unclear	L	L	Unclear	H
Li and Li (2018)	Unclear	Unclear	Unclear	Unclear	L	L	Unclear	H
Liang et al. (2020)	L	Unclear	Unclear	Unclear	L	L	L	L
Liu (2013)	L	Unclear	Unclear	Unclear	L	L	L	L
Luo et al. (2019)	L	Unclear	Unclear	Unclear	L	L	Unclear	L
Nie and Zou (2014)	Unclear	Unclear	Unclear	Unclear	L	L	L	L
Sun (2023)	L	Unclear	Unclear	Unclear	L	L	L	L
Wen (2012)	Unclear	Unclear	Unclear	Unclear	H	L	Unclear	H
Wen and Cao (2016)	L	Unclear	Unclear	Unclear	L	L	Unclear	L
Wu et al. (2023)	L	Unclear	Unclear	Unclear	L	L	Unclear	L
Xiao (2021)	L	Unclear	Unclear	Unclear	L	L	Unclear	L
Xue et al. (2024)	L	Unclear	Unclear	Unclear	L	L	L	L
Yang and Zhang (2009)	Unclear	Unclear	Unclear	Unclear	L	L	Unclear	H
Yang (2015)	Unclear	Unclear	Unclear	Unclear	H	L	Unclear	H
Ye (2010)	Unclear	Unclear	Unclear	Unclear	L	L	L	L
Zhang et al. (2022)	L	Unclear	Unclear	Unclear	L	L	Unclear	L
Zhang (2021)	L	Unclear	Unclear	Unclear	L	L	Unclear	L
Zhao (2020)	Unclear	Unclear	Unclear	Unclear	L	L	L	L

Abbreviations: L: low risk, H: high risk.

### 3.4 Meta-analysis

#### 3.4.1 Effective rate

**FIGURE 2:**
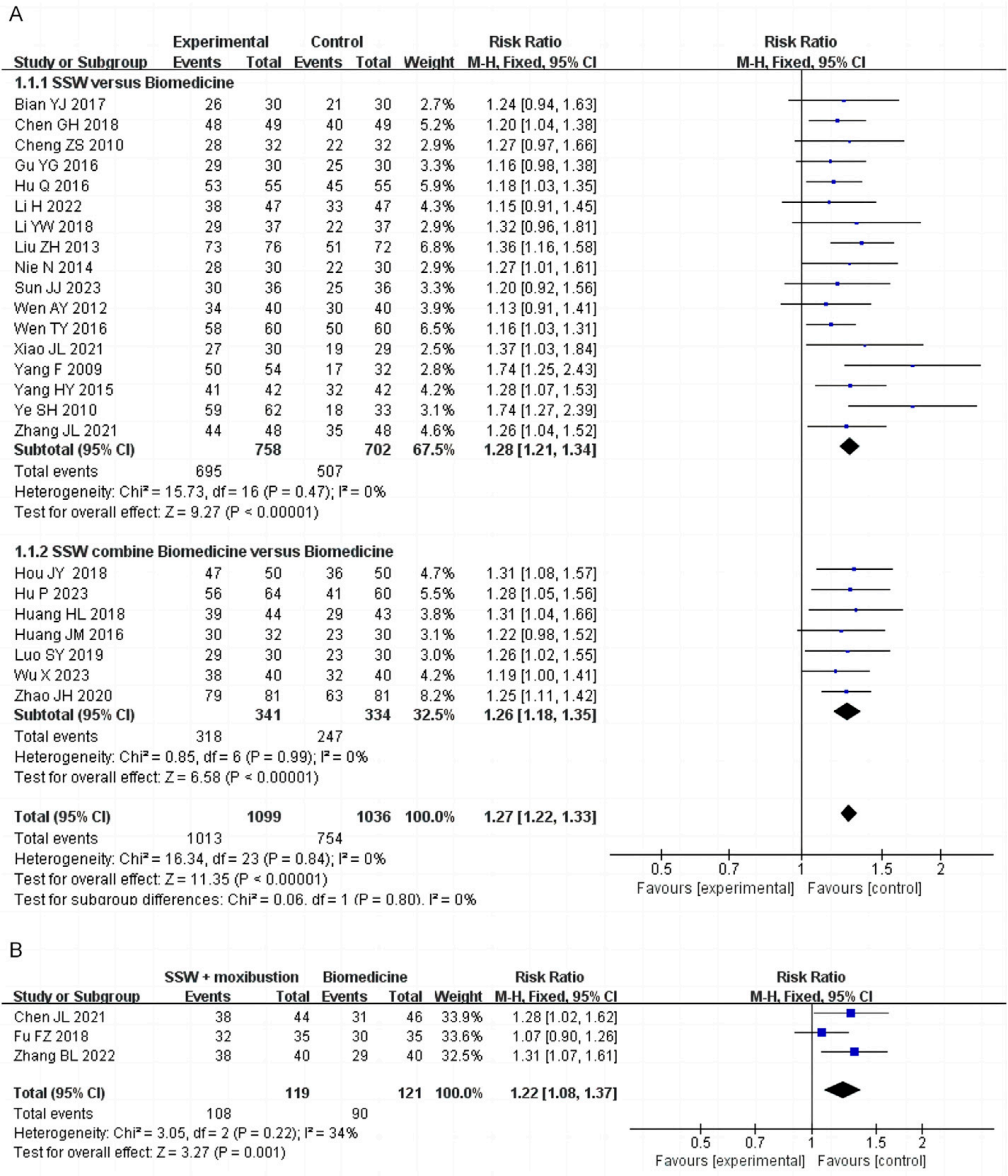
Forest plot of comparison of efficacy. **(A)** Forest plot of the efficacy of SSW for IBS‐D. **(B)** Forest plot of the efficacy of SSW combined with moxibustion for IBS‐D.

Twenty-four studies evaluated the efficacy of SSW in treating IBS-D, with 17 studies using SSW as the sole intervention and 7 studies using SSW combined with the same biomedicine as the control group. A total of 2,135 patients were included, comprising 1,099 in the treatment group and 1,036 in the control group. The pooled analysis showed low heterogeneity (I^2^ = 0%, P = 0.84); thus, a fixed-effects model was applied. The results indicated that SSW had a significantly higher effective rate compared to biomedicine (RR = 1.27; 95% CI: 1.22, 1.33; P < 0.00001). Subgroup analysis revealed that SSW was more effective than conventional biomedicine (RR = 1.28; 95% CI: 1.21, 1.34; P < 0.00001) and that combining SSW with biomedicine further improved efficacy (RR = 1.26; 95% CI: 1.18, 1.35; P < 0.00001) (Figure 2A).

Three studies assessed the efficacy of SSW combined with moxibustion, showing that this combination achieved better therapeutic outcomes than general biomedicine (RR = 1.22; 95% CI: 1.08, 1.37; P = 0.0001) (Figure 2B).

**FIGURE 3:**
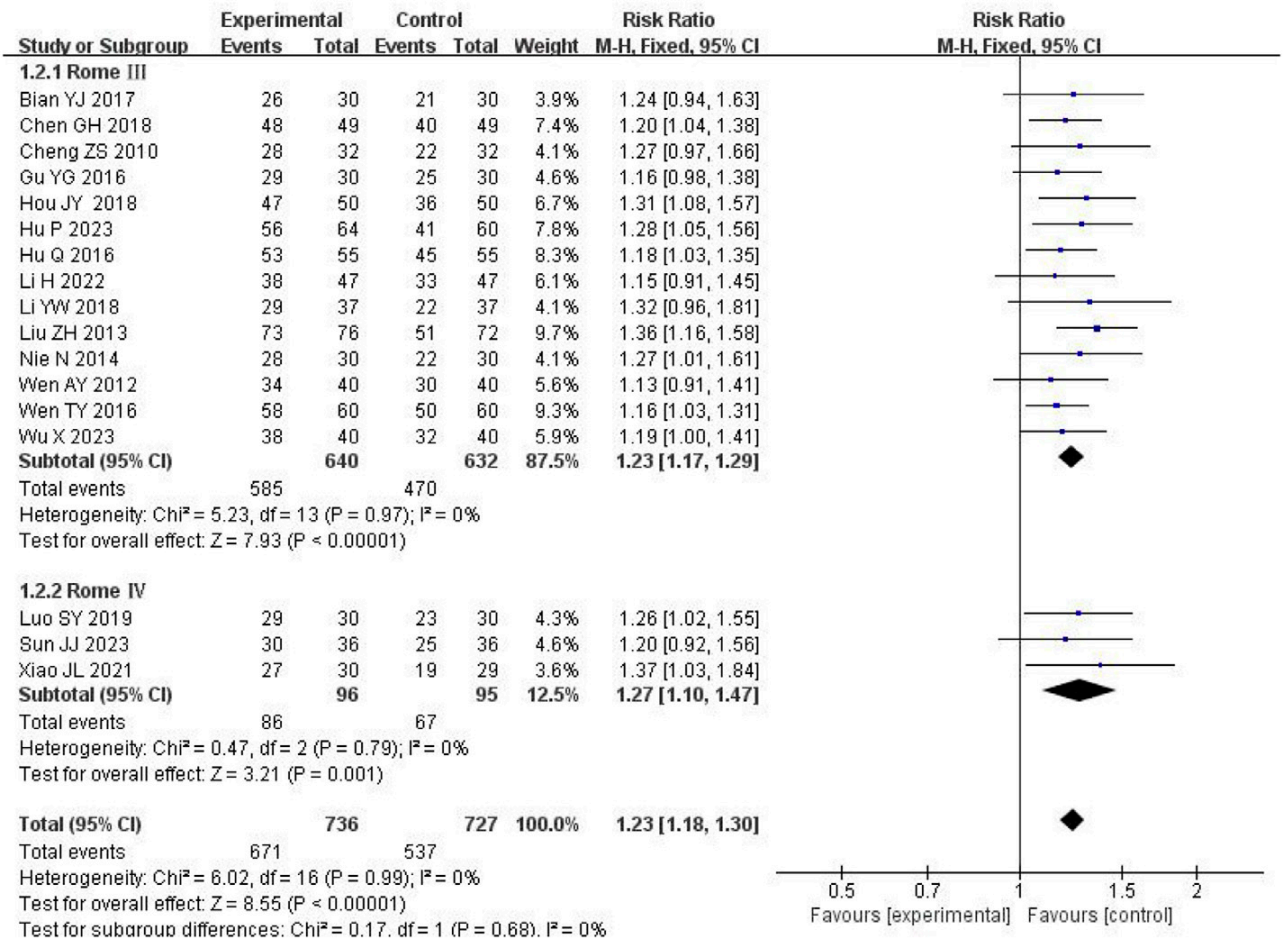
Forest plot of comparison of efficacy under different diagnostic criteria.

Of the studies reporting efficacy outcomes, 1 used Rome II criteria, 14 used Rome III as the diagnostic criteria, and 3 used Rome IV. To minimize potential bias arising from differing diagnostic criteria, we performed a subgroup analysis stratified by the diagnostic criteria. The pooled analysis showed low heterogeneity (I^2^ = 0%, P = 0.99); thus, a fixed-effects model was applied. Subgroup analysis demonstrated that SSW significantly enhanced the treatment efficacy for IBS-D based on both Rome III criteria (RR = 1.23; 95% CI: 1.17, 1.29; P < 0.00001) and Rome IV criteria (RR = 1.27; 95% CI: 1.10, 1.47; P = 0.001) (Figure 3).

#### 3.4.2 Symptom scores

**FIGURE 4:**
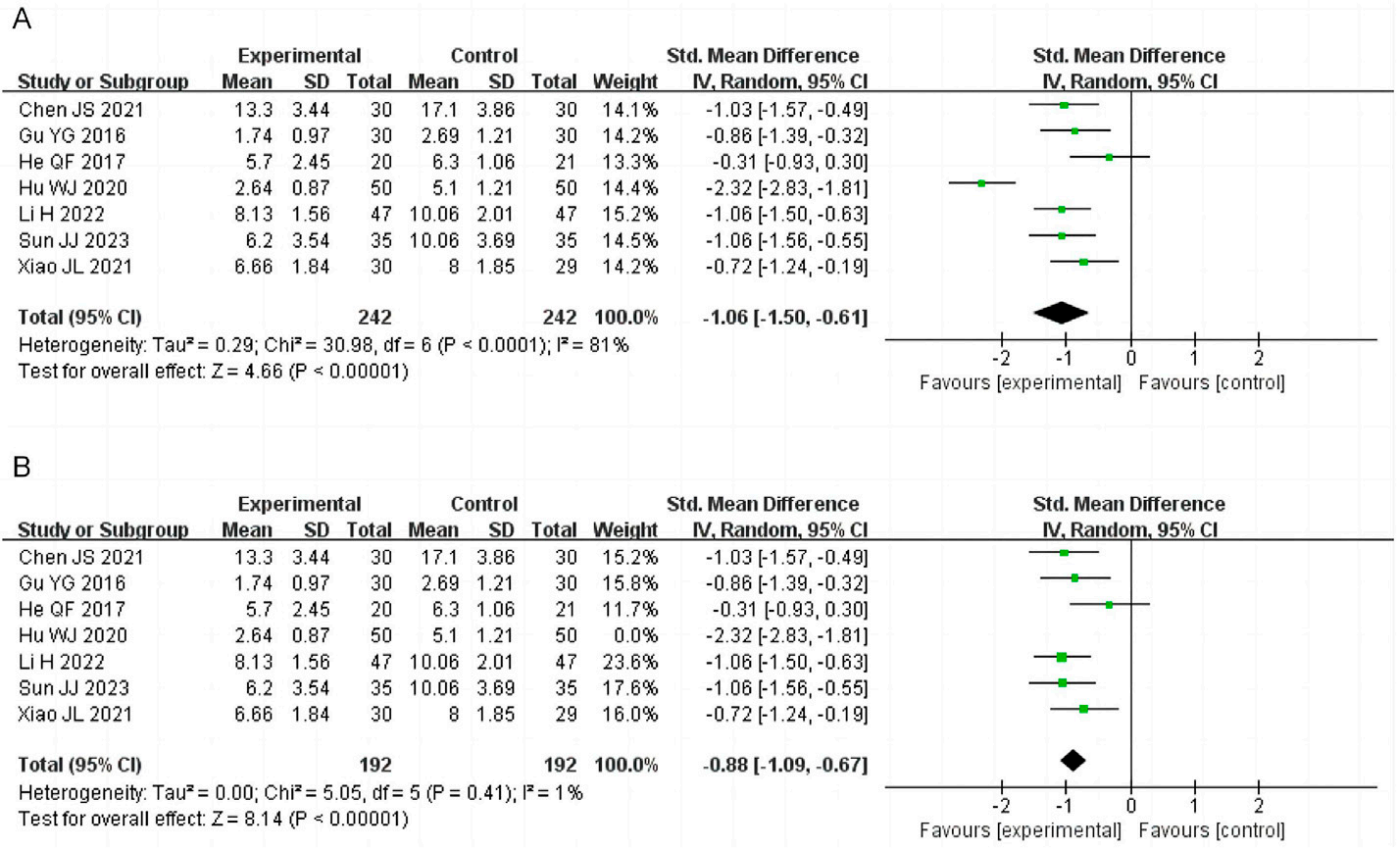
Forest plot of comparison of symptom scores. **(A)** Forest plot of comparison of symptom scores. **(B)** Sensitivity analysis of symptom scores.

Seven studies evaluated overall symptom scores, involving 484 patients (242 in the treatment group and 242 in the control group). High heterogeneity was observed (I^2^ = 81%, P < 0.0001), prompting the use of a random-effects model. The pooled results demonstrated that SSW significantly improved overall symptoms compared to biomedicine (SMD = −1.06; 95% CI: −1.50, −0.61; P < 0.00001) (Figure 4A). Sensitivity analysis identified the study by Hu WJ (2020) as the main source of heterogeneity. After its removal, heterogeneity decreased (I^2^ = 1%, P = 0.41), and the results remained consistent, indicating the robustness of the findings (SMD = −0.88; 95% CI: −1.09 to −0.67; P < 0.00001) (Figure 4B). Variations in clinical data collection may contributed to the observed heterogeneity.

#### 3.4.3 Abdominal pain score

**FIGURE 5:**
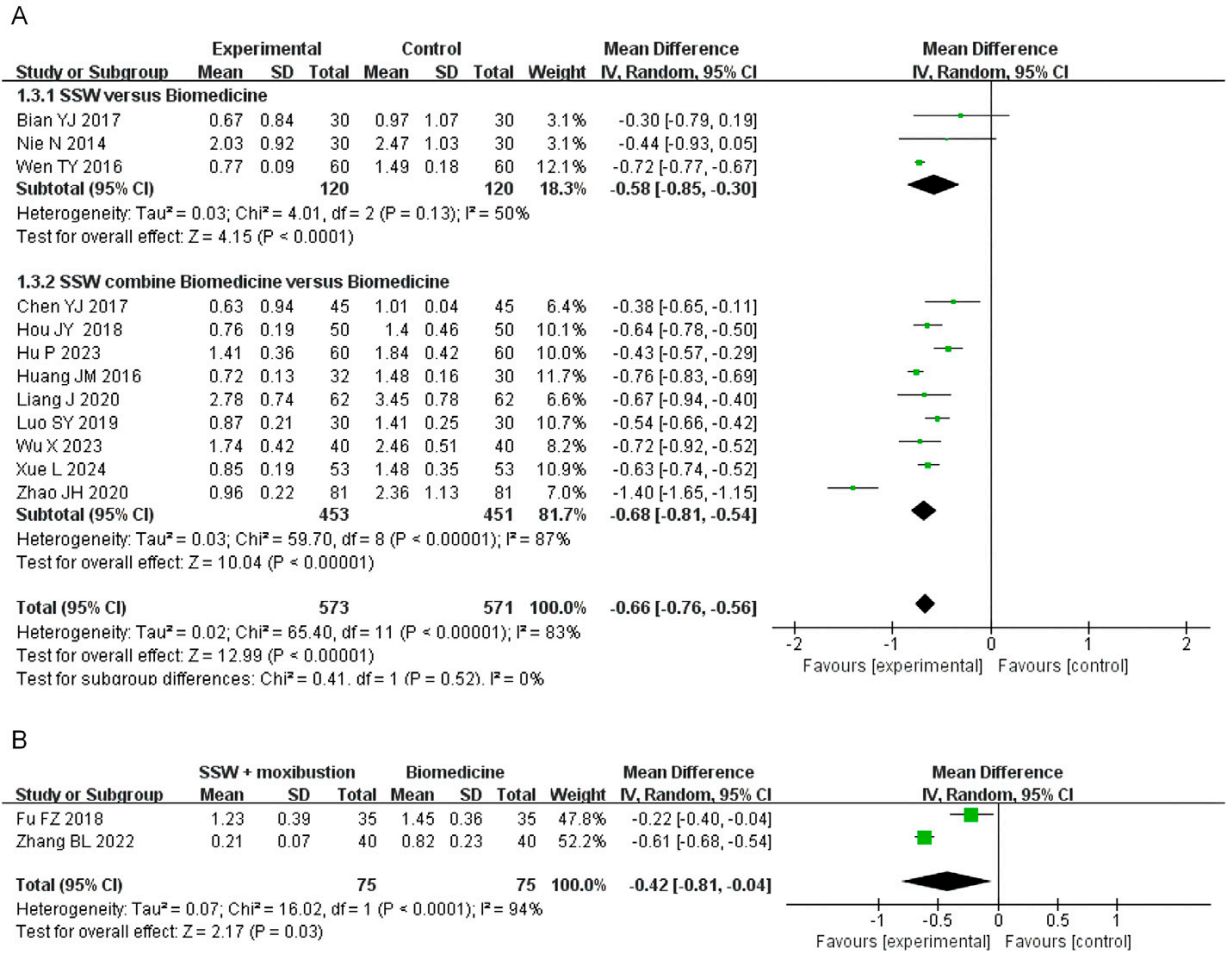
Forest plot of comparison of abdominal pain score. **(A)** Forest plot of the abdominal pain score of SSW for IBS-D. **(B)** Forest plot of the abdominal pain score of SSW combined with moxibustion for IBS-D.

Twelve studies reported abdominal pain scores, involving 1,144 patients. Due to high heterogeneity (I^2^ = 83%), a random-effects model was employed. SSW significantly alleviated abdominal pain symptoms (MD = −0.66; 95% CI: −0.76, −0.56; P < 0.00001). Subgroup analysis showed that SSW alone (MD = −0.58; 95% CI: −0.85, −0.30; P < 0.0001) and SSW combined with biomedicine (MD = −0.68; 95% CI: −0.81, −0.54; P < 0.00001) were both effective in reducing abdominal pain (Figure 5A). The sensitivity analysis was performed by removing the study in turn, and the combined effect did not change significantly. Therefore, the meta analysis results were relatively stable, considering the heterogeneity is caused by inconsistent research methods.

Two studies investigated the effect of SSW combined with moxibustion, demonstrating greater pain relief compared to biomedicine (MD = −0.42; 95% CI: −0.81 to −0.04; P = 0.03) (Figure 5B).

#### 3.4.4 Diarrhea score

**FIGURE 6:**
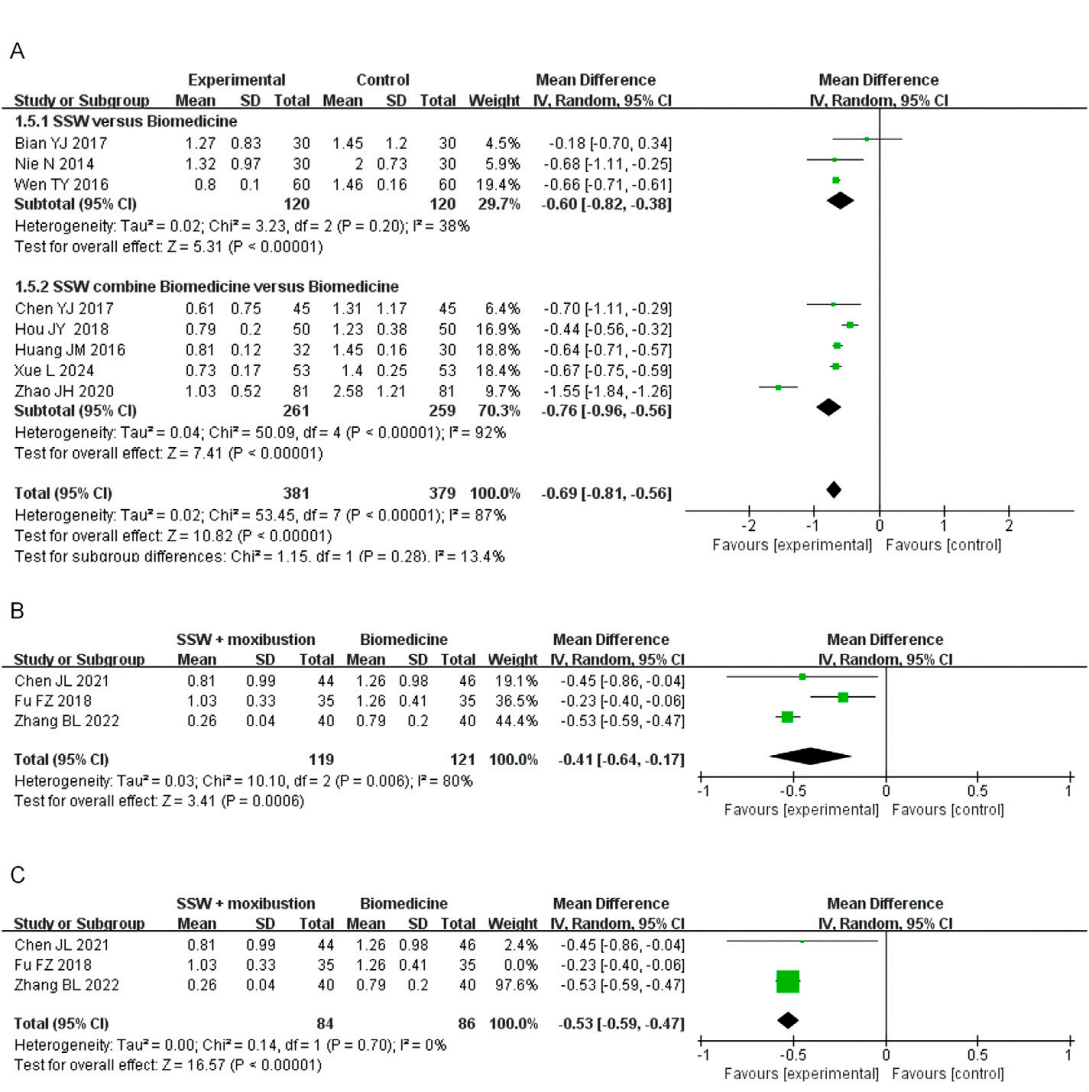
Forest plot of comparison of diarrhea score. **(A)** Forest plot of the diarrhea score of SSW for IBS-D. **(B)** Forest plot of the diarrhea score of SSW combined with moxibustion for IBS-D. **(C)** Sensitivity analysis of the diarrhea score of SSW combined with moxibustion for IBS-D.

Nine studies involving 760 patients assessed the effect of SSW on diarrhea symptoms. Moderate heterogeneity was observed (I^2^ = 87%, P < 0.00001), so a random-effects model was used. SSW significantly reduced diarrhea scores (MD = −0.69; 95% CI: −0.81, −0.56; P < 0.00001). Subgroup analysis indicated that SSW alone improved diarrhea symptoms (MD = −0.60; 95% CI: −0.82, −0.38; P < 0.00001) and enhanced the efficacy of biomedicine (MD = −0.76; 95% CI: −0.96, −0.56; P < 0.00001) (Figure 6A).

Three studies assessed SSW combined with moxibustion, revealing significant improvements (MD = −0.41; 95% CI: −0.64, −0.17; P = 0.0006) (Figure 6B). Sensitivity analysis identified Fu FZ (2018) as the primary source of heterogeneity. After excluding this study, heterogeneity decreased (I^2^ = 0%, P = 0.70), and results remained stable (MD = −0.53; 95% CI: −0.59, −0.47; P < 0.00001) (Figure 6C).

#### 3.4.5 Abdominal distension score

**FIGURE 7:**
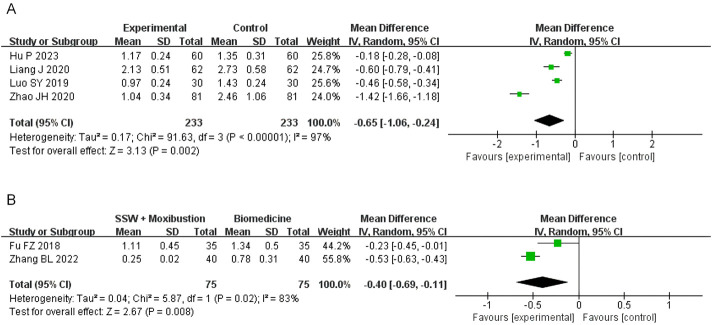
Forest plot of comparison of abdominal distension score. **(A)** Forest plot of the abdominal distension score of SSW for IBS-D. **(B)** Forest plot of the abdominal distension score of SSW combined with moxibustion for IBS-D.

Four studies involving SSW combined with biomedicine reported abdominal distension scores. High heterogeneity was observed (I^2^ = 97%, P < 0.00001), so a random-effects model was used. The results showed significant improvement in abdominal distension (MD = −0.65; 95% CI: −1.06, −0.24; P = 0.002) (Figure 7A). The sensitivity analysis was conducted by sequentially excluding individual studies, and the combined effect did not change significantly. Therefore, considering the heterogeneity arising from disparate research methodologies, the meta-analysis findings demonstrated relative stability.

Two studies evaluating SSW combined with moxibustion also reported significant improvements in abdominal distension symptoms (MD = −0.40; 95% CI: −0.69, −0.11; P = 0.008) (Figure 7B).

#### 3.4.6 Loss of appetite score

**FIGURE 8:**
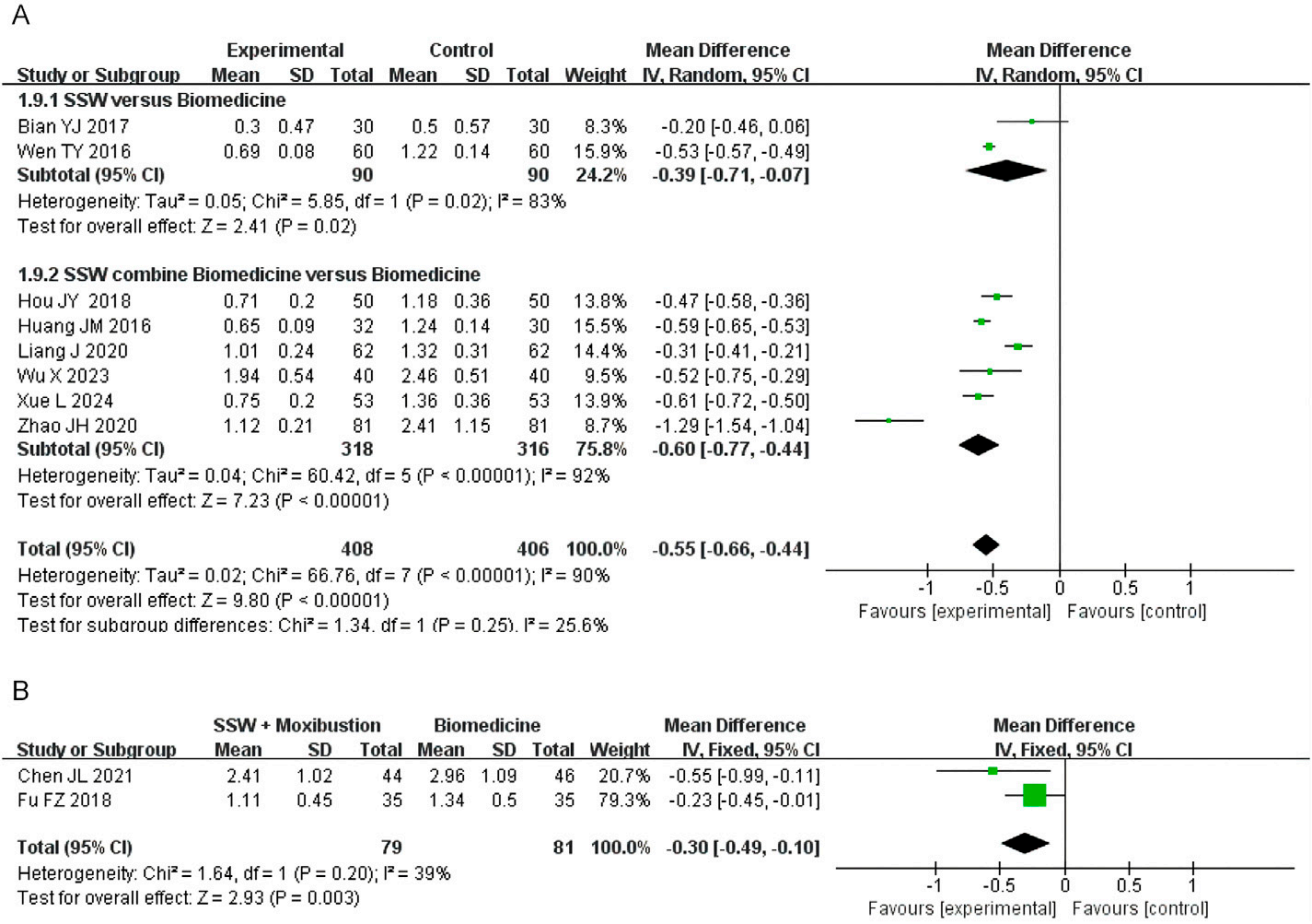
Forest plot of comparison of loss of appetite score. **(A)** Forest plot of the loss of appetite score of SSW for IBS-D. **(B)** Forest plot of the loss of appetite score of SSW combined with moxibustion for IBS-D.

Eight studies involving 814 patients evaluated loss of appetite, with 408 in the SSW treatment group and 406 in the control group. Due to high heterogeneity (I^2^ = 90%, P < 0.00001), a random-effects model was applied. SSW significantly improved appetite loss (MD = −0.55; 95% CI: −0.66, −0.44; P < 0.00001). Subgroup analysis showed that SSW alone (MD = −0.39; 95% CI: −0.71, −0.07; P = 0.02) and SSW combined with biomedicine (MD = −0.60; 95% CI: −0.77, −0.44; P < 0.00001) effectively reduced appetite loss (Figure 8A). The sensitivity analysis was performed by removing the study in turn, and the combined effect did not change significantly. Therefore, the meta analysis results were relatively stable, considering the heterogeneity is caused by inconsistent research methods.

Two studies on SSW combined with moxibustion demonstrated improvements in appetite (MD = −0.30; 95% CI: −0.49, −0.10; P = 0.003) (Figure 8B).

#### 3.4.7 Recurrence rate

**FIGURE 9:**
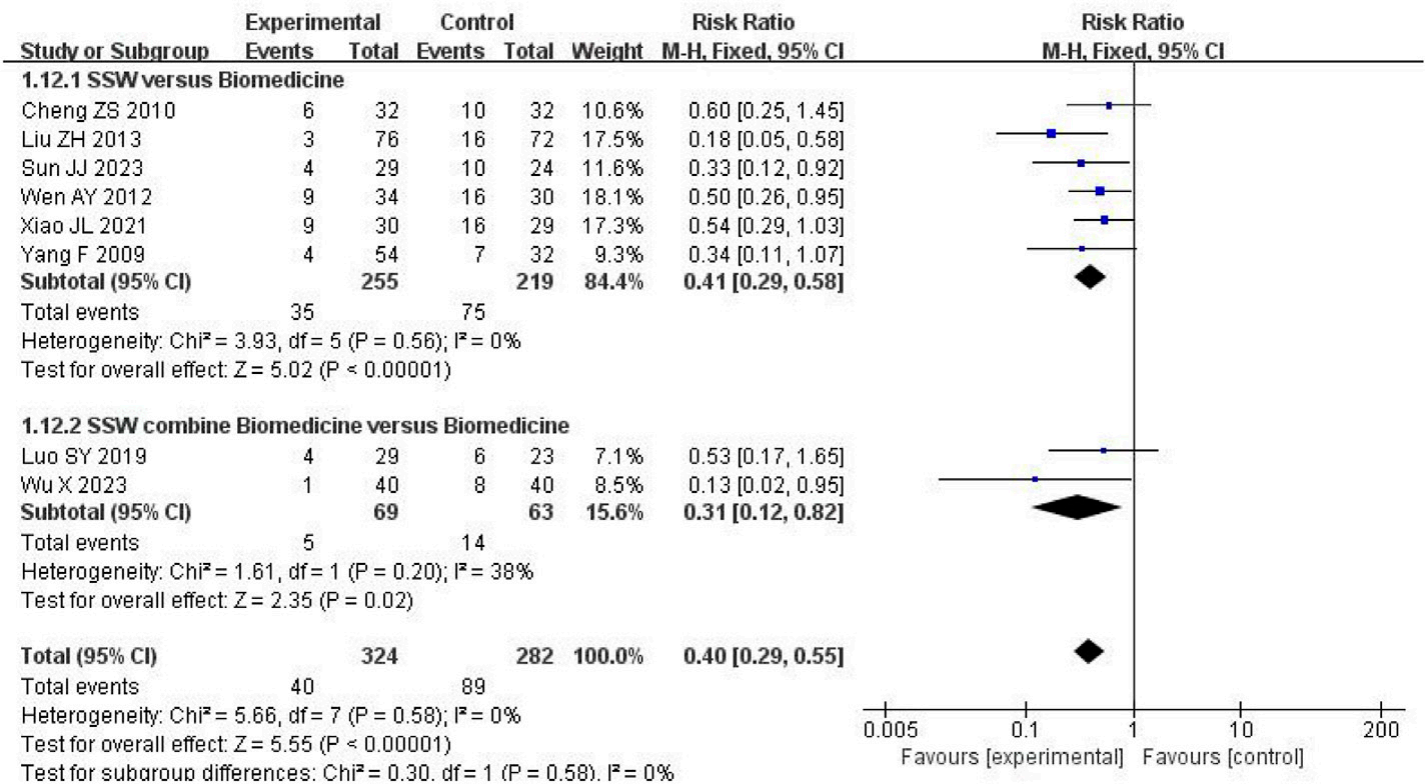
Forest plot of comparison of recurrence rate.

Eight studies reported recurrence rates, with follow-up periods ranging from 1 to 6 months. Among 324 patients treated with SSW, the recurrence rate was 12.35%, compared to 31.56% among 282 control group patients. Low heterogeneity was observed (I^2^ = 0%, P = 0.58), allowing for a fixed-effects model. The results showed that SSW significantly reduced recurrence rates (RR = 0.40; 95% CI: 0.29, 0.55; P < 0.00001). Subgroup analysis confirmed the efficacy of SSW alone (RR = 0.41; 95% CI: 0.29, 0.58; P < 0.00001) and SSW combined with biomedicine (RR = 0.31; 95% CI: 0.12, 0.82; P = 0.02), with reliable results (I^2^ = 0%) (Figure 9).

#### 3.4.8 Security analysis

Among 34 studies, 13 reported adverse reactions, while 21 did not mention them. Details are provided in Table 1. No adverse effects were found in 13 studies, while constipation (Chen et al., 2018; Fu et al., 2018; Liang et al., 2020; Xue et al., 2024), nausea (Chen, 2021; Huang, 2018; Xue et al., 2024; Zhao, 2020), dry mouth (Chen et al., 2018; Huang, 2018; Liang et al., 2020), insomnia (Huang, 2018; Liang et al., 2020), rash (Huang, 2018; Liang et al., 2020) and headache (Liang et al., 2020) were the main adverse effects mentioned in the other 7 studies.

### 3.5 Trial sequential analysis (TSA)

**FIGURE 10:**
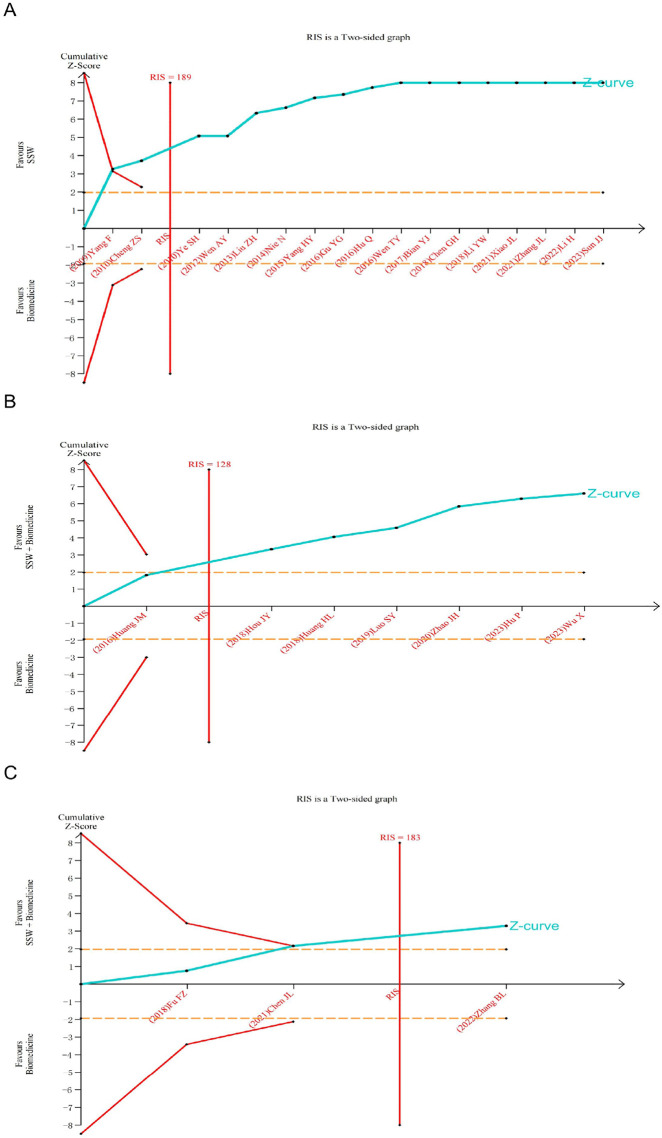
Trial sequential analysis for the effective rate in all included trials. **(A)** SSW as an adjuvant to biomedicine for IBS-D. **(B)** SSW combined with biomedicine as an adjuvant to biomedicine for IBS-D. **(C)** SSW combined with moxibustion as an adjuvant to biomedicine for IBS-D.

The TSA was further conducted based on the efficacy of SSW in treating IBS-D. Analyses were performed according to different interventions and diseases within the treatment group. As shown in Figure 10A, the cumulative Z-value crossed the TSA threshold, indicating that SSW alone is effective for IBS-D. Furthermore, the cumulative Z-value reached the required information size (RIS), suggesting that the sample size of the current study is sufficient. In contrast, Figure 10B shows that the cumulative Z-value crossed the traditional significance threshold but did not reach the TSA boundary, implying that more trials are needed to confirm the efficacy of SSW combined with biomedicine for IBS-D. Finally, Figure 10C demonstrates that the cumulative Z-value crossed the TSA threshold for SSW combined with moxibustion, confirming its effectiveness for IBS-D. The RIS was also achieved in this case.

## 4 Discussion

IBS-D is a prevalent functional gastrointestinal disorder with a complex pathogenesis involving brain-gut interaction disorders, psychological factors, abnormal intestinal motility, visceral hypersensitivity, dysbiosis, and genetic influences (Chey et al., 2015). These factors interact to contribute to IBS symptoms, as evidenced by a retrospective analysis of 407 patients, which highlighted the cumulative effects of visceral hypersensitivity, abnormal colonic transit, and psychological factors on gastrointestinal and non-gastrointestinal symptoms, as well as quality of life (Simrén et al., 2019). The onset and progression of IBS are influenced by a multitude of factors. Nonetheless, the pharmacological interventions frequently employed in clinical settings—including antispasmodic agents, antidiarrheal medications, gastrointestinal motility drugs, and anxiolytic and antidepressant therapies—often fail to produce satisfactory outcomes.

SSW, a TCM formula first recorded in the *Pu Ji Ben Shi Fang* of the Song Dynasty in China (Xu, 2007), is a classic prescription for diarrhea and is widely used for IBS-D. SSW comprises six botanical drug ingredients, including Myristica fragrans Houtt, Psoralea corylifolia, Schisandra chinensis, Tetradium ruticarpum, Zingiber officinale, Ziziphus jujuba. Studies have shown that SSW alleviates abdominal pain in IBS-D by regulating the p38 MAPK/JNK signaling pathway, reducing TRPV1 neuron activity, and mitigating visceral hypersensitivity (Li et al., 2024). Additionally, it protects the intestinal mucosal barrier by modulating the expression of tight junction proteins (Occludin and Claudin-1) and balancing pro-inflammatory (IL-6, IL-1β, TNF-α) and anti-inflammatory (IL-10) cytokines. It also enhances gastrointestinal function by increasing motilin and gastrin levels (Liu et al., 2019).

Network pharmacology analysis identified β-sitosterol, berberine, and stigmasterol as key active components of SSW (Shen and Guan, 2022). Among them, β-sitosterol and stigmasterol, plant sterols with anti-inflammatory properties, inhibit inflammatory pathways involving TNF-α, IL-6, IL-1β, and COX-2 through the NF-κB pathway, thereby reducing intestinal mucosal inflammation (Feng et al., 2018). Berberine, with calcium channel antagonist activity, reduces intestinal hypersensitivity and abnormal motility, contributing to IBS-D symptom relief (Chai et al., 2019).

Moxibustion, a common TCM external therapy, is also effective in treating IBS-D. Clinical studies demonstrate that moxibustion improves IBS-D symptoms, reduces rectal sensitivity, and enhances quality of life (Bao et al., 2016; Zhu et al., 2014). Animal studies suggest that its mechanisms include reducing visceral hypersensitivity, regulating gut microbiota, and attenuating inflammation (Zhao et al., 2016; Wang et al., 2016; Wang et al., 2018). Therefore, The combined use of SSW and moxibustion provides complementary benefits for IBS-D treatment.

This meta-analysis is the first to systematically review RCTs of SSW for IBS-D, incorporating TSA to estimate sample size. The results can provide a more objective evaluation of current research and a new level of evidence for patients, policymakers, and physicians. A total of 34 studies were included: 21 used SSW alone, 10 combined SSW with biomedicine, and 3 combined SSW with moxibustion. The findings indicate that SSW, either alone or in combination with other therapies, enhances treatment efficacy and reduces symptom scores compared to biomedicine. The efficacy of these treatments renders them a promising option, serving as a viable alternative or adjunctive therapy for individuals with IBS-D.

The majority of studies included in this investigation employed the Rome diagnostic criteria (including Rome Ⅱ, Rome Ⅲ and Rome Ⅳ). Previous studies have shown that the prevalence of IBS-D varies under different diagnostic criteria (Priya et al., 2020). To minimize potential confounding effects arising from heterogeneous diagnostic criteria, we performed stratified subgroup analyses based on diagnostic classification in the efficacy evaluation. The results showed that SSW could effectively improve the efficacy of IBS-D according to Rome Ⅲ and Rome Ⅳ diagnostic criteria, and the results were stable and reliable.

In 13 studies reporting safety data, 39 mild and tolerable adverse events were recorded in each of treatment and control groups. However, due to inconsistent reporting and the absence of routine safety assessments (e.g., blood tests, liver/kidney function), a meta-analysis of adverse events was not feasible. Nevertheless, no significant differences in adverse events between SSW and biomedicine were observed, indicating that SSW is safe for clinical use. Future trials should incorporate comprehensive safety assessments to strengthen the evidence base.

The recurrence of IBS-D remains a challenge. Among the included studies, eight reported follow-up data (1–6 months), demonstrating that SSW effectively reduces recurrence rates, further supporting its clinical utility.

The TSA results confirmed the effectiveness of SSW, either alone or combined with moxibustion, for IBS-D treatment, with the current sample size reaching the RIS, indicating no need for further sample expansion. This provides a strong foundation for recommending SSW as a therapeutic option for IBS-D. However, the efficacy of SSW combined with conventional treatments for IBS-D requires validation through additional RCTs. It is important to note that TSA cannot address the methodological quality issues present in the included RCTs, which may compromise the reliability of the results. Consequently, these findings should be interpreted with caution.

This study has several limitations. First, some included studies lacked detailed descriptions of their randomization methods. Second, information on allocation concealment and blinding was insufficient. In clinical trials, blinding is essential to reduce bias; however, in this included literature, no study used double-blind design. This limitation may stem from the inherent differences between TCM formulations and biomedicine, which make implementing blinding challenging. Nonetheless, the absence of blinding may introduce bias into the results. Third, the outcome indicators reported across the included studies were inconsistent, and there is potential publication bias due to the non-publication of negative results. Fourth, most of the studies were single-center trials with small sample sizes and limited long-term follow-up evaluations of treatment effectiveness. Consequently, rigorous multi-center studies with extended follow-up periods are needed for result verification. Lastly, as all participants in the included studies were Chinese, the generalizability of these findings to other ethnic groups is limited.

## 5 Conclusion

Compared with biomedicine alone, SSW alone or combined with biomedicine significantly improved treatment efficacy, reduced the overall symptom score, alleviated key symptoms (abdominal pain, diarrhea, abdominal distension, loss of appetite), and reduced the recurrence rate. SSW combined with moxibustion also improved treatment outcomes, reduced abdominal pain, diarrhea, abdominal distension and loss of appetite. No obvious adverse reactions were observed. However, methodological limitations persist in the existing evidence base, particularly regarding constrained sample sizes, insufficient follow-up periods, and the absence of double-blinded randomization procedures. These preliminary findings should be interpreted cautiously until replicated in adequately powered, double-blind RCTs with standardized outcome measures. Future research should focus on designing and conducting high-quality, long-term, randomized, double-blind clinical trials to further enhance the reliability and generalizability of the research findings.

## Data Availability

The original contributions presented in the study are included in the article/Supplementary Material, further inquiries can be directed to the corresponding author.
