# Supplementary material for: Efficacy and safety of Chinese botanical drug Si Shen Wan in irritable bowel syndrome: a meta-analysis and trial sequential analysis of randomized controlled trials
Source: Front Pharmacol. 2025 Jun 2;16:1534904. doi: 10.3389/fphar.2025.1534904 (PMC12171203; doi:10.3389/fphar.2025.1534904)
Supplement: Supplementary file 1 [file Supplementaryfile1.docx]

**Search strategy**

#1 (Irritable Bowel Syndromes) or (Irritable Bowel Syndromes

(Syndrome, Irritable Bowel) or (Syndromes, Irritable Bowel) or (Colon, Irritable) or (Irritable Colon) or (Colitis, Mucous) or (Colitides, Mucous) or (Mucous Colitides) or (Mucous Colitis)

#2 (si shen) or (si shen wan) or (si shen pill) or (sishen) or (sishen wan) or (sishen pill)

#3 (clinical trial) or (randomized controlled trial) or (randomised controlled trials).

Search：#1 and #2 and #3
